# HSPA8 acts as an amyloidase to suppress necroptosis by inhibiting and reversing functional amyloid formation

**DOI:** 10.1038/s41422-023-00859-3

**Published:** 2023-08-14

**Authors:** Erpeng Wu, Wenyan He, Chenlu Wu, Zhangcheng Chen, Shijie Zhou, Xialian Wu, Zhiheng Hu, Kelong Jia, Jiasong Pan, Limin Wang, Jie Qin, Dan Liu, Junxia Lu, Huayi Wang, Jixi Li, Sheng Wang, Liming Sun

**Affiliations:** 1 State Key Laboratory of Cell Biology, Center for Excellence in Molecular Cell Science, Chinese Academy of Sciences, University of Chinese Academy of Sciences, Shanghai, China; 2 State Key Laboratory of Molecular Biology, Center for Excellence in Molecular Cell Science, Chinese Academy of Sciences, Shanghai, China; Key Laboratory of Systems Health Science of Zhejiang Province, School of Life Science, Hangzhou Institute for Advanced Study, University of Chinese Academy of Sciences, Hangzhou, China; 3 School of Life Science and Technology, ShanghaiTech University, Shanghai, China; 4 State Key Laboratory of Genetic Engineering, School of Life Sciences and Huashan Hospital, Shanghai Engineering Research Center of Industrial Microorganisms, Fudan University, Shanghai, China

**Keywords:** Necroptosis, Cell signalling

## Abstract

Ultra-stable fibrous structure is a hallmark of amyloids. In contrast to canonical disease-related amyloids, emerging research indicates that a significant number of cellular amyloids, termed ‘functional amyloids’, contribute to signal transduction as temporal signaling hubs in humans. However, it is unclear how these functional amyloids are effectively disassembled to terminate signal transduction. RHIM motif-containing amyloids, the largest functional amyloid family discovered thus far, play an important role in mediating necroptosis signal transduction in mammalian cells. Here, we identify heat shock protein family A member 8 (HSPA8) as a new type of enzyme — which we name as ‘amyloidase’ — that directly disassembles RHIM-amyloids to inhibit necroptosis signaling in cells and mice. Different from its role in chaperone-mediated autophagy where it selects substrates containing a KFERQ-like motif, HSPA8 specifically recognizes RHIM-containing proteins through a hydrophobic hexapeptide motif N(X_1_)φ(X_3_). The SBD domain of HSPA8 interacts with RHIM-containing proteins, preventing proximate RHIM monomers from stacking into functional fibrils; furthermore, with the NBD domain supplying energy via ATP hydrolysis, HSPA8 breaks down pre-formed RHIM-amyloids into non-functional monomers. Notably, HSPA8’s amyloidase activity in disassembling functional RHIM-amyloids does not require its co-chaperone system. Using this amyloidase activity, HSPA8 reverses the initiator RHIM-amyloids (formed by RIP1, ZBP1, and TRIF) to prevent necroptosis initiation, and reverses RIP3-amyloid to prevent necroptosis execution, thus eliminating multi-level RHIM-amyloids to effectively prevent spontaneous necroptosis activation. The discovery that HSPA8 acts as an amyloidase dismantling functional amyloids provides a fundamental understanding of the reversibility nature of functional amyloids, a property distinguishing them from disease-related amyloids that are unbreakable in vivo.

## Introduction

Amyloids are a type of most well-known higher-order protein structures, all of which contain a central cross-β spine, with solvent-excluded, self-complementing steric zipper interactions. They were initially defined as a distinct type of protein folding disorder that causes unspecific cellular dysfunction in a variety of diseases, including Alzheimer’s disease. Surprisingly, in addition to playing devastating role in human diseases, amyloids have been found to contribute to functional entities in living organisms, such as HET-s mediating heterokaryon incompatibility and Sup35 suppressing inherited nonsense codon in fungus.^1^ In the early 2000s, a melanosome protein Pmel17 was found to form amyloid templates that facilitate melanin deposition during melanosome biogenesis in human melanocytes and pigment cells;^2,3^ thus the Pmel17 fibril is the first human proteinaceous fibril found to fit the term ‘functional amyloids’. Later, in 2009, several peptide-hormones were found to form amyloids stored in pituitary secretory granules.^4^ The term ‘functional amyloids’ was coined subsequently to distinguish these signaling amyloids from extracellularly deposited disease-related amyloids.^5,6^

In 2012, the amyloidal nature of a class of cytosolic RIP homotypic interaction motif (RHIM)-containing proteins, receptor-interacting serine/threonine-protein kinase 1 (RIP1) and 3 (RIP3), were unveiled.^7^ In the following years, other RHIM-amyloids, ZBP1 (also known as DNA-dependent activator of interferon regulatory factors (DAI) or DLM-1) and Toll/IL-1R domain-containing adapter-inducing interferon-β (TRIF) are sequentially confirmed to share the similar amyloidal characteristic.^8,9^ RHIM-based amyloids are currently the largest functional amyloid family in mammals, and all these RHIM-containing proteins are involved in necroptosis signal transduction.^10^

Necroptosis is a pro-inflammatory membrane-lytic cell death, which is mediated by the membrane-damaging protein mixed lineage kinase domain-like pseudokinase (MLKL). The formation of multi-level RHIM-amyloids is required for necroptosis signal propagation to MLKL.^7–10^ There are three initiator RHIM-containing proteins, each responding to different extracellular stimuli, to initiate necroptosis signaling in mammalian cells:^11^ RIP1 transmits the signal from the TNF family of cytokines; ZBP1 responds to zRNA/zDNA; TRIF responds to TLR3/4. The initiator RHIM-containing proteins form amyloidal seeds and recruit their shared downstream effector RHIM-containing protein, RIP3, via the RHIM–RHIM homophilic interaction. More cytosolic RIP3 monomers were then nucleated to form RIP3 fibrils.^12–16^ RIP3 fibril elongation causes oligomerization and activation of MLKL, resulting in necroptotic membrane rupture.^17,18^ In summary, RHIM-based amyloids function as macromolecular signaling hubs to activate MLKL.

The RHIM motif is a conserved sequence of ~18 amino acids shared by the four mammalian RHIM-containing proteins for necroptosis signal transduction. However, one nature law should not be ignored: signal transduction is a temporal process, and therefore no signaling hub should be permanently assembled. In the cell-free system, the RHIM-core, with a conserved tetrad peptide sequence (I/V/L)-(Q/M)-(I/V/L)-G, spontaneously nucleates RHIM monomers to form rigid fibrils, and the amyloidal fibrils keeps growing due to adjacent cross-β-sheet packing, which creates an energy barrier for dissociation.^6,19–21^ However, in live cells, neither spontaneous RHIM-amyloid growth nor necroptosis occurs, despite the fact that RHIM-containing proteins are expressed at high levels in certain tissues such as the spleen and duodenum.^22^ Furthermore, after the necroptosis inducer was removed, cells were even able to survive via ESCRT complex-mediated membrane repair, despite in the presence of already formed RHIM-amyloids.^23^ It is unknown how cells suppress spontaneous RHIM-amyloid formation or resolve fibrous polymers to avoid necroptosis.

Here we identify heat shock protein family A member 8 (HSPA8, also called HSC70 or HSP70-8) as the first amyloidase in mammalian cells, with the specific role of reversing RHIM-amyloids to prevent necroptosis by serving a dual role: (1) blocking spontaneous RHIM oligomerization to stop fibril growth, and (2) disassembling pre-formed RHIM-fibrils in an ATP-dependent manner. Therefore, the RHIM-amyloids emerge as a class of legitimate substrates for the amyloidase HSPA8 to restrain functional amyloid-mediated signal transduction.

## Results

### HSPA8 suppresses necroptosis

**Fig. 1:**
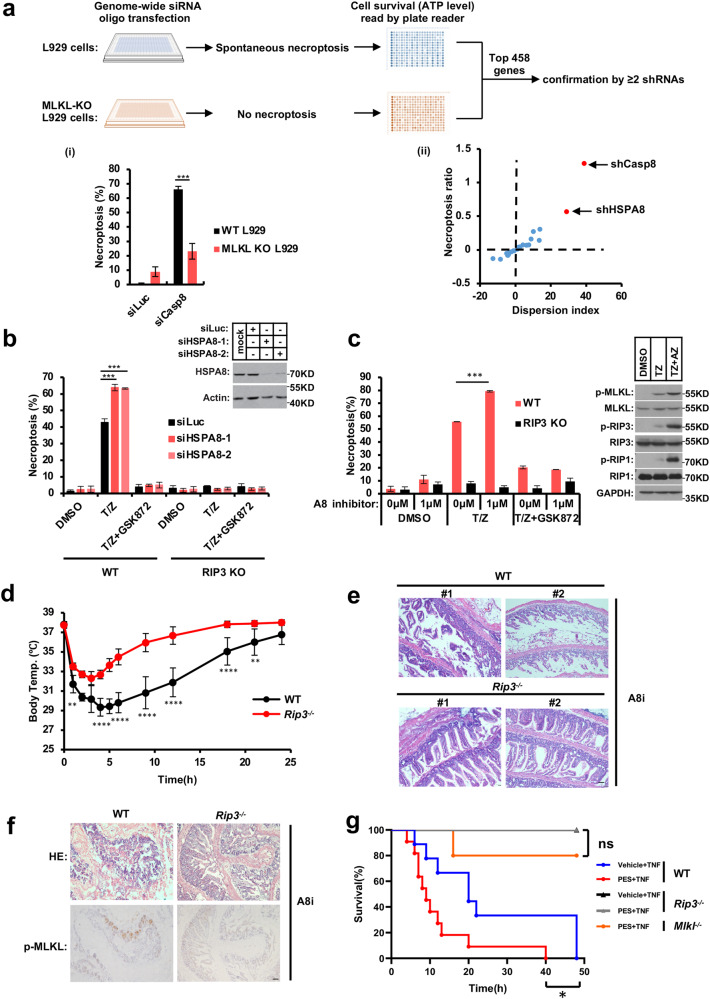
HSPA8 suppresses necroptosis. **a** *Hspa8* was identified as a necroptosis-inhibitory gene by a genome-wide siRNA screening. WT and MLKL-KO L929 cells were seeded in 384-well plates and transfected with siRNA pools in parallel. After 72 h, cell viability was determined by measuring intracellular ATP levels as described in Materials and methods. The hit siRNA oligos (targeting 458 genes) were ranked, and the top 23 genes were further confirmed individually with two or more lentivirus-shRNAs (listed in Supplementary information, Fig. S1a) as shown in scatter plot (ii). siCaspase-8 was the positive control that induced spontaneous necroptosis in the WT L929 but not in the MLKL-KO L929 cells, as shown in (i). **b** Knocking down HSPA8 promoted RIP3-dependent necroptosis in mouse L929 cells. HSPA8 was knocked down by siRNA transfection for 36 h, and necroptosis was induced by treating cells with T/Z for 3 h. The siHSPA8-enhanced necroptosis was blocked by treating cells with RIP3 kinase inhibitor GSK872 (2 mM) or knocking out RIP3. Two oligos, siHSPA8-1 and siHSPA8-2, were used to confirm the necroptosis-inhibitory role of HSPA8 by knocking down the expression of HSPA8. Cell viability was determined by measuring intracellular ATP levels. Necroptosis inducer T/Z: T, TNFα (20 ng/mL); Z, z-VAD (20 μM). The data are represented as mean ± SD of duplicate wells. The knockdown efficiency of HSPA8 was tested by immunoblotting analysis (right panel). **c** Inhibition of HSPA8 promoted necroptosis signaling. The WT L929 cells and RIP3-KO L929 cells were pretreated with HSPA8 inhibitor AZ for 2 h. Then necroptosis was induced by treating cells with T/Z for 3 h. Cell viability was determined by measuring intracellular ATP levels. The data are represented as mean ± SD of duplicate wells. The necroptosis molecular markers (p-RIP1, p-RIP3, and p-MLKL) were analyzed with the indicated antibodies by immunoblotting (right panel). **d** HSPA8 inhibitor injection induced hypothermia. The WT and *Rip3*^*−/−*^mice were injected with HSPA8 inhibitor PES (90 mg/kg). Body temperature was measured at the indicated intervals. *n* = 6 mice for each group. **e** Representative intestinal histological images of WT and *Rip3*^*−/−*^ mice after HSPA8 inhibitor injection. WT and *Rip3*^*−/−*^ mice were treated with the HSPA8 inhibitor PES (90 mg/kg) once daily for two consecutive days. Afterward, the intestines were collected and fixed in 4% paraformaldehyde at 4 °C for 48 h. Paraffin sections were prepared following standard protocols, and hematoxylin was used for counterstaining. Scale bar, 50 μm. **f** Representative immunohistochemistry images of intestine tissue showing the p-MLKL signal in WT mice after HSPA8 inhibitor injection. The WT and *Rip3*^*−/−*^mice were injected with HSPA8 inhibitor PES (90 mg/kg) as detailed in **e**. Scale bar, 50 μm. **g** The Kaplan–Meyer survival curve showed that HSPA8 protected TNF-induced SIRS. Before TNF (7 μg) injection, the WT, *Rip3*^*−/−*^, and *Mlkl*^*−/−*^ mice were pre-injected with vehicle control or HSPA8 inhibitor PES (45 mg/kg) for 30 min. Mouse survival was monitored at 120-min intervals. *n* ≥ 5 mice for each group. *P* values were determined by unpaired two-tailed Student’s *t*-test with Welch’s correction. ns, no significance. *P < 0.05; **P < 0.01; ***P < 0.005. All results are reported from one representative experiment from at least three independent repeats.

Necroptosis is tightly suppressed in vivo to prevent overt inflammation.^24–26^ We found that silencing the expression of Caspase-8, a proteinase that cleaves RIP1 and RIP3,^27–29^ caused spontaneous necroptosis in wild-type (WT) L929 cells, and that knocking out MLKL, the key membrane-damaging protein for necroptosis, prevented siCaspase-8-induced necroptosis (Fig. 1a(i)). Therefore, we set up a genome-wide RNA interference screen, using a synthetic small interfering RNA (siRNA) library and siCaspase-8 oligo as a positive control, to identify necroptosis inhibitory genes. Each of the top hit siRNAs was independently confirmed with more than two different small hairpin RNAs (shRNAs) (Supplementary information, Fig. S1a). Aside from shCaspase-8, the top-one hit-shRNA against HSPA8 expression induced spontaneous necroptosis in WT L929 cells, which could be protected by *Mlkl* deficiency (Fig. 1a(ii)). Re-expressing an siRNA-resistant form of HSPA8 (HSPA8-scr) restored HSPA8’s necroptosis-inhibitory function (Supplementary information, Fig. S1b). In comparison to other members of the heat shock protein superfamily, only HSPA8 significantly inhibits necroptosis (Supplementary information, Fig. S1c), implying that this unique regulation is not shared by other members of the heat shock protein superfamily.

L929 cells are highly sensitive to TNF-induced necroptosis and capable of autocrine TNF signaling under stress conditions.^30,31^ Next, we investigated whether the increased necroptosis caused by HSPA8 knockdown was dependent on TNF signaling and whether TNF autocrine signaling was involved. TNFR1 knockout did not prevent siHSPA8-induced spontaneous necroptosis in L929 cells (Supplementary information, Fig. S1d); additionally, ablation of HSPA8 expression or application of HSPA8 inhibitor further enhanced necroptosis induced by recombinant TNF overload (Fig. 1b, c), and overexpression of HSPA8 suppressed recombinant TNF-induced necroptosis (Supplementary information, Fig. S1e). According to these findings, HSPA8 did not interfere with TNFR activation on plasma membrane or extracellular TNF autocrine signaling per se, but specifically hijacked the intracellular signal transduction of necroptosis. Furthermore, knocking down HSPA8 expression substantially increased both TNF- and TRAIL-induced necroptosis (Supplementary information, Fig. S1i). In addition, knocking down HSPA8 expression increased TLR3-, TLR4-, and ZBP1-initiated necroptosis in both mouse L929 and human HeLa-RIP3 cells (Supplementary information, Fig. S2a–f). These data implied that HSPA8 targets the shared component(s) of necroptosis pathways regardless of the extrinsic inducers.

The phosphorylations of RIP1, RIP3, and MLKL are markers for TNF-induced necroptosis. Inhibiting HSPA8 increased RIP1, RIP3, and MLKL phosphorylations after necroptosis induction (Fig. 1c, right panel). HSPA8 knockdown-enhanced cell death could be prevented by knocking out RIP3 or inhibiting RIP3 kinase activity with GSK872 (Fig. 1b), or by MLKL inhibitor necrosulfonamide (NSA) (Supplementary information, Fig. S1g, i). Consistently, HSPA8 inhibitor-enhanced necroptosis could also be blocked by *Rip3* deficiency or RIP3 kinase inhibitor (Fig. 1c). Re-introducing HSPA8-scr reversed the enhanced necroptosis by siHSPA8 (Supplementary information, Fig. S1b, h). In contrast, knocking down HSPA8 expression did not affect TNF-induced apoptosis in the *Rip3*-null HeLa cells or the *Mlkl*-knockout L929 cells (Supplementary information, Fig. S2g, h).

HSPA8 has been reported to play an important role in chaperone-mediated autophagy (CMA).^32,33^ To test whether HSPA8’s necroptosis-inhibitory role is also dependent on the CMA process, we knocked down the expression of LAMP2A, the HSPA8 downstream lysosomal receptor of the CMA machinery,^34^ and found that necroptosis in neither human HeLa-RIP3 nor the mouse L929 cells were affected (Supplementary information, Fig. S3). In addition, RIP1, RIP3, and MLKL all lack the KFERQ-like peptide motif required for HSPA8 recognition as CMA substrates. Together, HSPA8 suppressed necroptosis in a CMA-independent manner.

### HSPA8 inhibition induces necroptosis-mediated toxicity in mice

HSPA8 inhibitors have attracted intensive studies for treatment of cancer and autoimmune diseases, but such efforts were hindered by their side effects such as their toxicity to normal tissues.^35,36^ To test whether RIP3-dependent necroptosis contributes to the adverse effects of HSPA8 inhibitors, we treated the WT and *Rip3*^*−/−*^ mice with HSPA8 inhibitor Pifithrin-μ (PES) or Apoptozole (AZ). The WT mice developed hypothermia after receiving a peritoneal injection of PES, but this effect was reduced in the *Rip3*^*−/−*^ and *Mlkl*^*−/−*^ mice (Fig. 1d; Supplementary information, Fig. S4a, b). PES severely disrupted the crypts and villi, and caused massive immune cell infiltration into the small intestine and colon in WT mice; however, in the *Rip3*^*−/−*^ and *Mlkl*^*−/−*^ mice, this effect was significantly reduced (Fig. 1e; Supplementary information, Fig. 2c–e). Massive phosphorylated MLKL (p-MLKL) signals were detected in the damaged intestine epithelium of WT mice (Fig. 1f), indicating that PES treatment activated the necroptosis pathway. The p-MLKL signal was abolished in the intestine of *Rip3*^*−/−*^ and *Mlkl*^*−/−*^ mice (Fig. 1f; Supplementary information, Fig. S4f). These findings implied that HSPA8 inhibition caused RIP3-dependent necroptosis and offered light on the possibility of using simultaneous necroptosis inhibition to reduce the toxicity and side effects of HSPA8 inhibitor therapy in patients with autoimmune disorders or cancer.

**Fig. 2:**
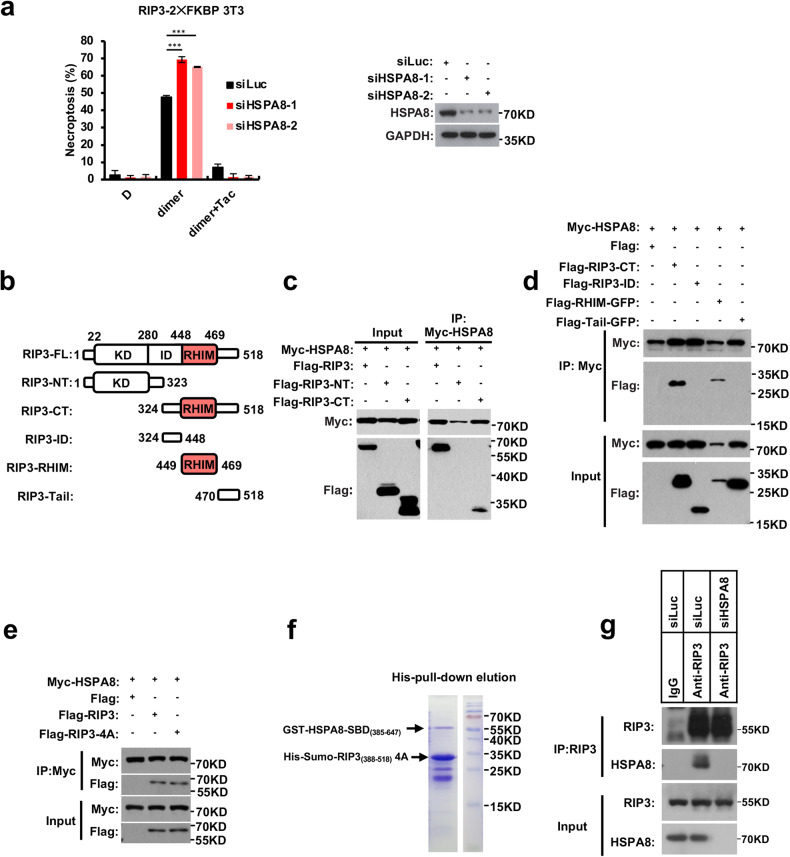
HSPA8 targets RIP3 to prevent the execution of necroptosis. **a** HSPA8 blocked RIP3 oligomerization-induced necroptosis. After 36 h of transfection of siHSPA8 oligos into the mRIP3-2×FKBP-3T3 cells, RIP3 oligomerization was induced by adding 20 nM FKBP dimerizer (AP20187, C_82_H_107_N_5_O_20_). The dimerizer competitor Tac (10 μM) was used as a negative control to block RIP3 oligomerization-induced necroptosis. The HSPA8 knockdown efficiency was tested by immunoblotting (right panel). **b** Schematic representation of the full-length and truncation variants of RIP3. FL, full-length; NT, N-terminal; CT, C-terminal; KD, kinase domain; ID, intermediate domain; RHIM, RIP homotypic interaction motif. **c,** **d** Mapping the HSPA8 binding region of RIP3 by co-immunoprecipitation. The Flag-tagged truncated RIP3 (as shown in **b**) and Myc-tagged HSPA8 cDNAs were co-transfected into 293FT cells. After 24 h of transfection, the whole-cell lysates were immunoprecipitated with anti-Myc beads, and immunoblotted with the indicated antibodies. Results are reported from one representative experiment from at least three independent repeats. **e** Mutations of the RHIM-core of RIP3 did not disrupt the interaction with HSPA8. Flag-tagged WT or the RHIM-core mutant, RIP3-4A mutant (VQVG into AAAA), was co-transfected with Myc-tagged HSPA8 into 293FT cells. Immunoprecipitation with anti-Myc beads was carried out as described in **c** and **d**. **f** In vitro His pull-down assay verified the interaction between HSPA8 and RIP3-RHIM. GST-tagged HSPA8-SBD domain (GST-HSPA8-SBD_385–647_) and His-tagged RIP3-RHIM region with the mutation of the RHIM-core (His-Sumo-RIP3_388–518_-4A) were purified from *E. coli* separately and mixed at the ratio of 1:1 (50 μM each). The His-tagged RIP3-RHIM was used as bait; the GST-tagged HSAP8 was used as prey and was pulled down by His-tagged RIP3-RHIM. **g** Co-immunoprecipitation of endogenous RIP3 and HSPA8. The L929 cells were transfected with the indicated siRNAs. After 48 h, the whole-cell lysate was incubated with anti-RIP3 antibody and protein A/G beads. The immunoprecipitated complexes were probed with the indicated antibodies. *P* values were determined by unpaired two-tailed Student’s *t*-test with Welch’s correction. **P < 0.01; ***P < 0.005. All results are reported from one representative experiment from at least three independent repeats.

It is widely accepted that necroptosis plays an essential role in systemic inflammatory response syndrome (SIRS).^24,37^ In line with the findings on the necroptosis-promoting role of HSPA8 inhibitor, pharmacological application of HSPA8 inhibitor synergistically increased SIRS-induced lethality in WT mice, but these effects were reduced in *Rip3*^*−/−*^ and *Mlkl*^*−/−*^ mice (Fig. 1g). *Rip3* deficiency also protected mice from HSPA8 inhibitor-enhanced intestinal damage (Supplementary information, Fig. S4g). In conclusion, HSPA8 inhibition exacerbated SIRS shock by increasing RIP3-dependent necroptosis. This study emphasized the importance of using HSPA8 inhibitors with caution in the context of cytokine storms, which can occur following systemic infections like sepsis and immunotherapies.

### HSPA8 targets RIP3 to prevent the execution of necroptosis

RIP3 activation is required for necroptosis execution by phosphorylating the membrane-damaging protein MLKL. We then investigated whether HSPA8 interferes with RIP3- or MLKL-mediated signal propagation. Since RIP3 and MLKL activation is dependent on their self-oligomerization, we developed a small molecule-induced protein oligomerization system (2×FKBP module-conjugated RIP3 or MLKL) to specifically initiate necroptosis by inducing RIP3 or MLKL oligomerization. The FKBP dimerizer, AP20187 (C_82_H_107_N_5_O_20_), is a synthetic and cell-permeable Tacrolimus (Tac or FK506) analog that acts as a chemical inducer of the FKBP-fusion protein dimerization. Tac, an FKBP dimerizer (AP20187) competitor, can be used as a negative control for the FKBP-dimerization system. Interestingly, knocking down the expression of HSPA8 enhanced cell death caused by RIP3 but not MLKL activation (Fig. 2a; Supplementary information, Fig. S5a).

To test whether HSPA8 recognizes RIP3, we co-expressed HSPA8 with full-length or truncated RIP3, as shown in Fig. 2b, and performed co-immunoprecipitation analysis. The antibody that immunoprecipitated HSPA8 also precipitated the full-length RIP3 (RIP3-FL), and the C-terminal RIP3 (RIP3-CT) containing the intermediate domain (RIP3-ID), the RIP3 RHIM motif (RIP3-RHIM), and RIP3 tail region (RIP3-Tail). The N-terminal RIP3 (RIP3-NT) containing kinase domain could not be co-precipitated with HSPA8 (Fig. 2c). We further truncated RIP3-CT into the intermediated domain, the RHIM-motif, and the tail region, and found that only the RHIM-motif could be co-precipitated with HSPA8 (Fig. 2d).

The VQI(V)G tetra-peptide core of RHIM motif is responsible for RIP3 packing and is buried in the center of the fibril. Interestingly, the alanine substitution of the tetra-peptide core (RIP3-4A) did not affect the interaction between RIP3 and HSPA8 (Fig. 2e), although it completely blocked RIP3-dependent necroptosis (Supplementary information, Fig. S5b). Such interaction was reconstituted in the cell-free system. The recombinant GST-tagged HSPA8 and the His-tagged RHIM-4A-containing RIP3 (388–518 aa) were separately purified from *E. coli*. The HSPA8-RIP3 mixture was purified using Ni-NTA affinity column after incubation. The resin captured both the His-tagged RIP3-RHIM-4A and the GST-tagged HSPA8 (Fig. 2f), indicating that HSPA8 recognized the RHIM-motif of RIP3 independently of the VQVG tetra-peptide core of RHIM motif. Endogenous immunoprecipitation also confirmed the interactions between HSPA8 and RIP3 in the living cells (Fig. 2g).

### HSPA8 inhibits RIP3-amyloid fibrillation in vitro and in cells

**Fig. 3:**
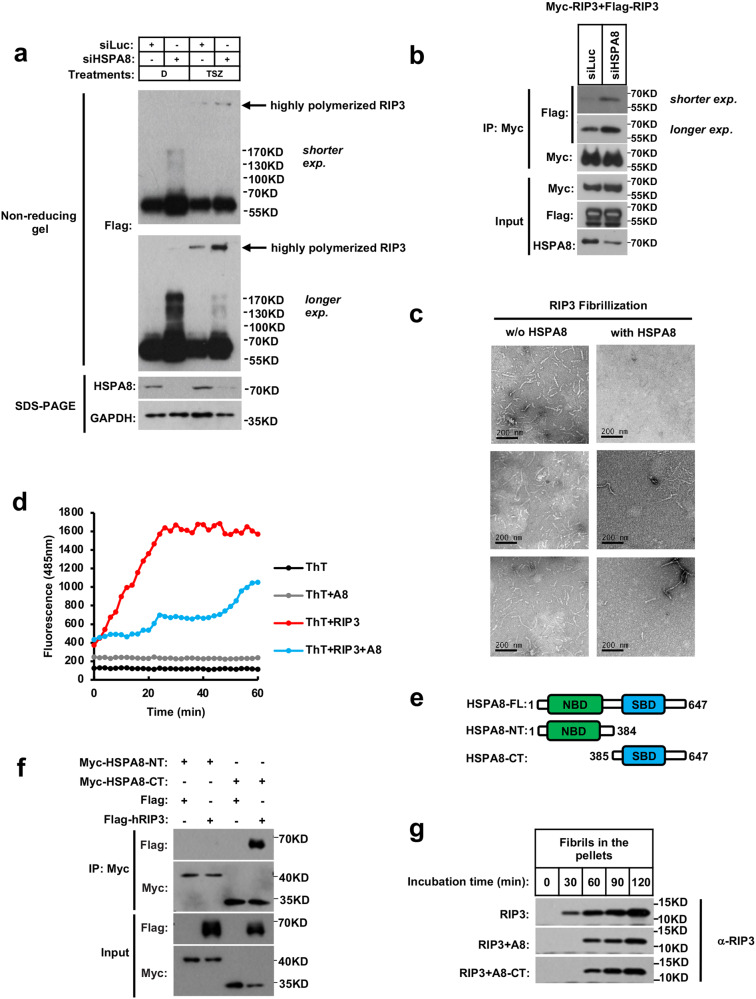
HSPA8 inhibits RIP3-amyloid fibrillation in vitro and in cells. **a** HSPA8 inhibited RIP3 spontaneous polymerization in vivo. Flag-RIP3-expressing HeLa cells were transfected with HSPA8 siRNA. Necroptosis was induced for 10 h. Whole-cell lysates were subjected to either non-reducing gel electrophoresis (to detect RIP3 polymerization as pointed by arrows) or SDS-PAGE (as loading control) as detailed in Materials and methods. RIP3 and HSPA8 levels were analyzed by immunoblotting with the indicated antibodies. GAPDH served as the loading control. **b** HSPA8 blocked RIP3 self-interaction in vivo. HeLa cells were co-transfected with Myc-tagged RIP3 and Flag-tagged RIP3, followed by knocking down the expression of HSPA8. Thirty-six hours later, the whole-cell lysates were subjected to immunoprecipitation with anti-Myc beads. The co-immunoprecipitated RIP3 was verified by immunoblotting. **c** Representative negative stain EM of newly formed RIP3 fibrils. HSPA8 and RIP3_(418–518)_ were purified from *E. coli* system. RIP3 fibril growth assay was carried out by incubating the fibrillation ‘seed’ RIP3 with freshly purified RIP3 monomers in the presence or absence of HSPA8 as described in Materials and methods. In short, the fibrillation ‘seeds’ (0.5 μM) were prepared by sonication of the pre-formed RIP3_(418–518)_ fibrils, and then incubated with freshly purified RIP3_(418–518)_ monomers (5 μM) in the presence or absence of HSPA8 (5 μM) at 37 °C for 2 h. The newly formed fibrils were precipitated for EM observation. Scale bars, 200 nm. **d** The growth process of RIP3 fibrils was monitored by ThT staining assay. The newly assembled RIP3 fibrils (as described in **c**) were stained with ThT (50 μM). The fluorescence intensity was measured every 2 min during the fibril growth process. Emission wavelength: 485 nm; excitation wavelength: 430 nm. **e** Schematic representation of the truncation strategy of HSPA8. NBD, nucleotide-binding domain; SBD, substrate-binding domain. FL, full-length; NT, N-terminal; CT, C-terminal. **f** Mapping of HSPA8 domain that mediated the interaction with RIP3. Myc-tagged truncated HSPA8 (as shown in **e**) was co-transfected with Flag-tagged RIP3 into 293FT cells. Twenty-four hours after transfection, the whole-cell lysates were subjected to immunoprecipitation with anti-Myc beads. The co-precipitated RIP3 and HSPA8 were quantified by immunoblotting with the indicated antibodies. Results are reported from one representative experiment from at least three independent repeats. **g** The SBD domain of HSPA8 is sufficient for blocking RIP3 fibril growth. RIP3 fibril growth was carried out in the presence of HSPA8-FL or HSPA8-CT as described in **c**. The newly formed RIP3 fibrils were pelleted and quantified by immunoblotting. Results are reported from one representative experiment from at least three independent repeats.

The RHIM motif drives RIP3 packing into fibrils after necroptosis induction via extensive Cys–Cys disulfide bond formation.^38^ Surprisingly, HSPA8 knockdown led to formation of polymeric RIP3 ladders even without necroptosis induction, as detected by non-reducing gel electrophoresis (Fig. 3a). When the expression of HSPA8 was silenced, the RIP3 monomers spontaneously assembled into oligomers as determined by co-immunoprecipitation (Fig. 3b).

Next, we performed an in vitro RIP3 fibril growth assay to test whether HSPA8 directly inhibits RIP3 amyloid stacking. The RIP3 fibril ‘seeds’ were prepared by sonicating the pre-formed RIP3 fibrils into nuclei, which were then incubated with soluble RIP3 monomers for fibrillation. The newly formed RIP3 fibrils were pelleted and examined by negative-stain electron microscopy (EM). The fibrillation process was monitored by time course of Thioflavin T (ThT) staining, a technique that was the most widely used “gold standard” for selectively staining amyloid fibrils. In the absence of HSPA8, RIP3 formed tubular fibrils; in the presence of HSPA8, no RIP3 fibril was formed (Fig. 3c). RIP3 fibrils grew rapidly and reached a peak value at 30 min without HSPA8; in the presence of HSPA8, the growth rate of RIP3 fibrils was significantly decreased (Fig. 3d).

HSPA8 consists of the N-terminal nucleotide binding domain (NBD) that possesses ATPase activity and the C-terminal substrate binding domain (SBD) for substrate recognition (Fig. 3e). Next, we set to map out the RIP3-binding region of HSPA8 and test whether the ATPase activity of HSPA8 is required to prevent RIP3-RHIM fibril growth. We co-expressed truncated HSPA8 with Flag-tagged RIP3 and found that only the SBD domain of HSPA8 co-immunoprecipitated with RIP3 (Fig. 3f). We then purified the HSPA8 SBD domain and incubated it with RIP3 for the fibrillation assay. The HSPA8 SBD domain suppressed RIP3 fibrillation as sufficiently as the full-length HSPA8 (Fig. 3g). Thus, we inferred that the binding of HSPA8-SBD to RIP3 was sufficient to sterically hinder the intermolecular association of RIP3 and prevent its amyloid formation, which is independent of the ATPase activity of HSPA8.

### HSPA8 disassembles the pre-formed RIP3 amyloidal fibrils coupled with ATP hydrolysis

**Fig. 4:**
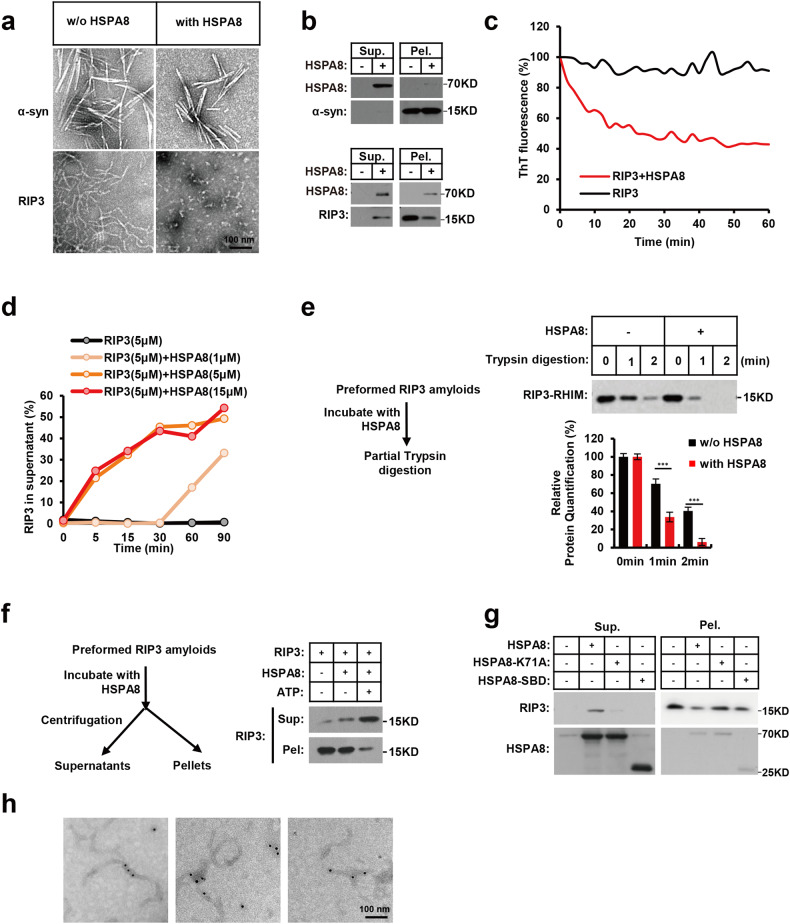
HSPA8 disassembles the pre-formed RIP3 amyloidal fibrils coupled with ATP hydrolysis. **a** Representative EM analysis of HSPA8-mediated disassembly of RIP3 fibrils. The pre-formed α-syn (upper panels) or RIP3 (bottom panels) fibrils were incubated with HSPA8 (5 μM), along with the ATP regeneration system, and ATP (4 mM) for 2 h, and analyzed by negative stain EM as detailed in Materials and methods. Scale bar, 100 nm. **b** Immunoblotting analysis of the disassembled RIP3 fibrils. After the disassembly reaction as described in **a**, the supernatant fraction (the disassembled soluble proteins) and pellet fraction (the insoluble fibrils) were separated by centrifugation and analyzed by immunoblotting with the indicated antibodies. **c** The disassembly process of RIP3 fibrils was monitored by ThT staining assay. The disassembled RIP3 fibrils (described in **a**) were stained with ThT (50 μM). The fluorescence intensity was measured every 2 min during the disassembly process; the ThT signals of sonicated fibrils were subtracted as baseline signals from the ThT time course data. The collected data were normalized with the ThT fluorescence intensity of the initial RIP3 fibrils before the disassembly reaction. Excitation wavelength: 430 nm; emission wavelength: 485 nm. **d** The stoichiometric ratio of the HSPA8 to RIP3 in the disassembly reaction system. The curves showed the real-time kinetics of the disassembled RIP3 fibrils in the presence of different ratios of HSPA8. **e** Partial trypsin digestion analysis of the aggregated RIP3 fibrils. Left: assay design of partial trypsin digestion. After the disassembly reaction, the RIP3 fibrils were incubated with trypsin (50 μg/mL) at 37 °C for the indicated times. The undigested RIP3 was detected by immunoblotting with anti-RIP3 antibody (upper panel). Lower chart: quantification of the RIP3 signals in the upper panel by image J. **f** ATP is required for the amyloidase activity of HSPA8. Left: Assay design of analyzing the RIP3 fibrils by centrifugation. RIP3 fibrils were disassembled by incubating with HSPA8 (as described in **a**) in the presence or absence of ATP. After the disassembly reaction, the disassembled RIP3 and the insoluble fibrils were separated by centrifugation and analyzed by immunoblotting with the indicated antibodies. **g** The ATP hydrolysis activity of HSPA8 is required for its amyloidase activity. The WT full-length, C-terminal fragment (385–647aa), and the full-length ATPase-deficient mutant (K71A) of HSPA8 were purified from *E. coli* and incubated with the pre-formed RIP3 fibrils, respectively. After the disassembly reaction, the soluble RIP3 and the insoluble fibrils were separated by centrifugation and analyzed by immunoblotting with the indicated antibodies. **h** Negative staining immunogold EM of HSPA8 binding to RIP3 fibrils. The microscope images shown here are representative examples of HSPA8-SBD bound to RIP3 fibrils. Anti-HSPA8 antibody was used at a dilution of 1:100. The binding of the antibody was visualized by adding an anti-rabbit gold-conjugated antibody. All results are reported from one representative experiment from at least three independent repeats.

In addition to their fast fibrillation, amyloid structures are highly stable and difficult to disassemble. Even after 30 days of co-incubation, HSPA8 alone cannot disaggregate the unfolded Parkinson’s disease-linked presynaptic protein α-synuclein (α-syn) fibrils.^39,40^ Surprisingly, after incubation with HSPA8, the RIP3 fibrils were quickly dismantled into a small globular structure representing small RIP3 oligomers disassembled from the mature fibrils, as shown in EM images in Fig. 4a. The resolved fibrils in the supernatant were centrifuged and examined by immunoblotting (Fig. 4b).

ThT staining revealed that in the absence of HSPA8, RIP3 amyloids exhibited stable ThT emission at ~485 nm, whereas in the presence of HSPA8, pre-formed RIP3 fibrils were disassembled within 40 min (Fig. 4c). The kinetics of HSPA8 amyloidase activity at different concentrations was then measured. Given that HSPA8 needs to prevent the disassembled monomer RIP3 from re-forming fibrils, we speculated that the stoichiometric ratio of HSPA8 to RIP3 in the disassembly assay should not be less than 1:1. As expected, the amyloidase activity of HSPA8 began to emerge only when the stoichiometric ratio of HSPA8 to RIP3 reached 1:1 (Fig. 4d).

**Fig. 5:**
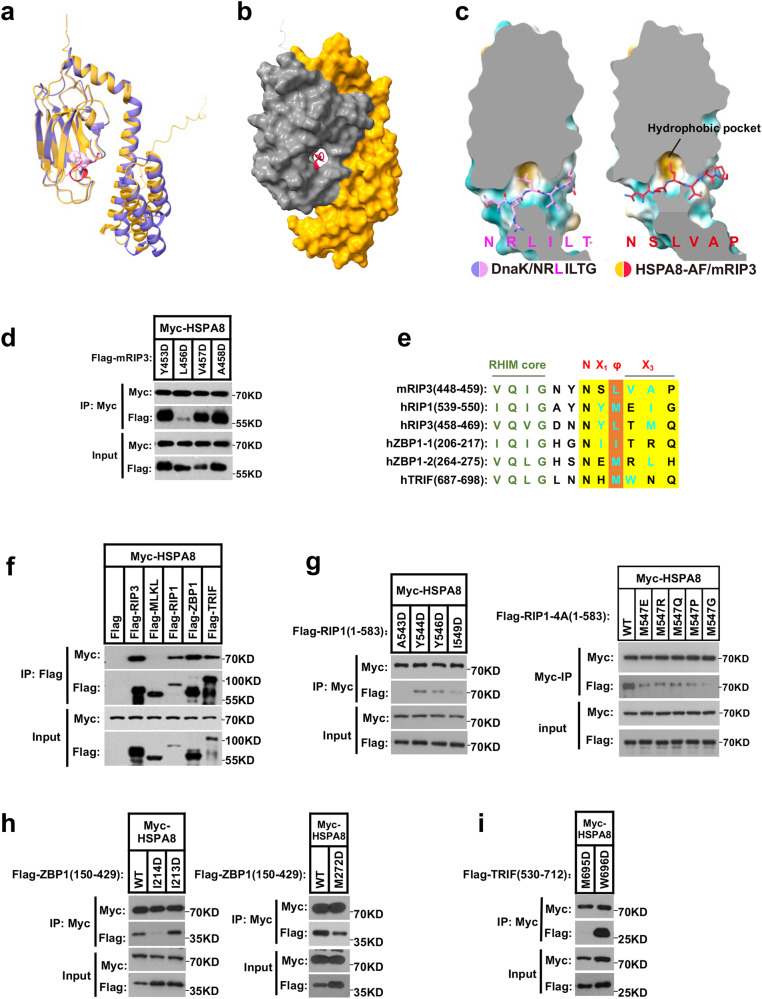
A consensus hexapeptide motif of the RHIM-containing proteins is required for HSPA8 recognition. **a** Comparison of the peptide substrate recognition modes in SBD of HSPA8 (orange) and DnaK (purple). HSPA8 β-subdomain bound mouse RIP3 peptide NSLVAP (red); DnaK bound its substrate peptide NRLILT (pink). Structure of the DnaK/NRLILT complex referenced in PDB (ID: 4EZY). Model of the HSPA8/NSLVAP complex derived from AlphaFold prediction. **b** Surface view of the mRIP3 peptide bound to the HSPA8-SBD hydrophobic pocket. Gray, β-subdomain; orange, α-subdomain. **c** The hydrophobic pockets of DnaK (left) and HSPA8 (right), and the bound peptides. **d** The hydrophobic residues of mouse RIP3 hexapeptide are required for the interaction with HSPA8. The hydrophobic residues of the mRIP3 hexapeptide were individually mutated to aspartic acid (D). Flag-tagged mutant RIP3 was co-transfected with Myc-tagged mHSPA8 into 293 T cells. The whole-cell lysates were subjected to immunoprecipitation with anti-Myc beads. **e** Sequence alignment of hexapeptides of the RHIM-containing proteins. mRIP3: mouse RIP3 (448–459aa); hRIP1: human RIP1 (539–550aa); hRIP3: human RIP3 (458–469aa); hZBP1-1: the first RHIM motif of human ZBP1 (206–217aa); hZBP1-2: the second RHIM motif of human ZBP1 (264–275aa); hTRIF: human TRIF (687–698aa). The RHIM-core tetrapeptide was highlighted in green; the hydrophobic residues within the hexapeptides were colored in cyan. **f** Interactions between HSPA8 and the initiator RHIM-containing proteins. The Myc-tagged HSPA8 and the individual Flag-tagged RHIM cDNAs were co-transfected into 293 T cells. Twenty-four hours after transfection, the whole-cell lysates were subjected to immunoprecipitation using anti-Flag beads and immunoblotted with the indicated antibodies. Results are reported from one representative experiment from at least three independent repeats. **g**–**i** The hydrophobic residues in hexapeptides of the RHIM-containing proteins were required for the interaction with HSPA8. The hydrophobic residues in hexapeptides of the RHIM-containing proteins were individually mutated (**g** for RIP1, **h** for ZBP1, and **i** for TRIF) and co-transfected with Myc-tagged HSPA8 into 293 T cells. The whole-cell lysates were subjected to immunoprecipitation with anti-Myc beads.

Interestingly, it is commonly observed that the stoichiometric ratios of enzyme to substrate are often less than 1:1. However, HSPA8, acting as an “amyloidase”, performs a dual role in both the pellet and the supernatant fractions, necessitating a higher total amount of HSPA8. Firstly, HSPA8 catalyzes the disassembly of RHIM-containing proteins in the pellet (Figs. 4b, g, 7c). Secondly, HSPA8 acts as a chaperone, binding to the non-fibril RHIM monomers in the supernatant to prevent their reassembly (Figs. 2c–e, 3f, 5d, f–i). The amount of HSPA8 in the pellet fraction is significantly lower than in the supernatant fraction, suggesting that the energy-consuming catalytic function of HSPA8 is highly efficient. However, the disassembled RHIM monomers require a larger quantity of HSPA8 to inhibit their reassembly, and this process is independent of ATP hydrolysis. Therefore, to maintain the RHIM-containing protein in its fully disassembled state, a 1:1 stoichiometric ratio of HSPA8 to RHIM is necessary.

To further support this, we performed a fibril disassembly assay by co-incubating RIP3 with equimolar HSPA8-SBD and varying concentrations of HSPA8-FL. The SBD fragment lacks the enzymatic capacity for fibril disassembly but retains the ability to prevent the reassembly of RIP3 monomers. Notably, when the stoichiometric ratio of HSPA8-FL to RIP3 was reduced to 1:10, the disassembled RIP3 was hardly detected. However, in the presence of HSPA8-SBD, a significant amount of RIP3 was disassembled and stabilized in the supernatant (Supplementary information, Fig. S6c, upper panel). As the stoichiometric ratio of HSPA8-FL to RIP3 increased to 0.4:1, the increment of disassembled RIP3 by the presence of SBD-HSPA8 diminished (Supplementary information, Fig. S6c, middle panel). Furthermore, when the stoichiometric ratio of HSPA8-FL to RIP3 reached 1:1, the presence of SBD-HSPA8 did not further facilitate RIP3 disassembly (Supplementary information, Fig. S6c, bottom panel). These data demonstrate that the amyloidase enzymatic activity of HSPA8 is highly efficient, and that HSPA8 must engage the monomers of RIP3 to prevent their reassembly.

To evaluate the aggregating status of RIP3 fibrils after incubation with the HSPA8 amyloidase, we measured the sensitivity of RIP3 fibril and HSPA8-disassembled RIP3 to limited trypsin proteolysis. The less exposed RIP3 buried in the fibrillous structure survived trypsin digestion and could be detected by immunoblotting with anti-RIP3 antibody after a series of controlled short exposures to limited trypsin digestion. The HSPA8-disassembled RIP3 was less compact than the pre-formed RIP3 fibrils and was less resistant to trypsin digestion (Fig. 4e).

To verify whether the amyloidase activity of HSPA8 depends on ATP, we incubated the RIP3 fibrils with HSPA8 and an ATP regeneration system with or without ATP. RIP3 fibrils were rapidly disassembled by HSPA8 in the presence of ATP and shifted into the supernatant fraction; in the absence of ATP, RIP3 fibrils remained intact and were retained in the pellet fraction (Fig. 4f). The requirement of ATP hydrolysis for HSPA8-mediated RIP3 fibril disassembly was further confirmed with an ATP hydrolysis-deficient mutant HSPA8-K71A.^41^ Neither the K71A mutant nor the C-terminal SBD domain of HSPA8 could disassemble RIP3 fibrils (Fig. 4g). Immunogold EM staining also confirmed that HSPA8 binds to RIP3 fibrils (Fig. 4h).

As expected, the endogenous necrosome RIP3–MLKL complex was disassembled following HSPA8 treatment (Supplementary information, Fig. S6a). Furthermore, MLKL was recruited by the disassembled RIP3, exhibiting reduced oligomerization (Supplementary information, Fig. S6b). It is worth noting that the oligomerization of MLKL is reliant on the formation of RIP3 fibrils, which plays a critical role in necroptosis signaling.^16^ Therefore, our data indicate that the disassembly of RIP3 fibrils, triggered by HSPA8 treatment, leads to the release or breakdown of MLKL oligomers.

### A consensus hexapeptide motif of the RHIM-containing proteins is required for HSPA8 recognition

The structure of HSPA8’s prokaryotic analog DnaK in complex with its substrate peptide has been determined.^42,43^ Based on molecular modeling and force field calculations by AlphaFold, we predicted HSPA8 SBD-binding mRIP3-RHIM peptide stretch using structural information from DnaK-SBD binding with its substrate peptide ‘NRLILT’. A hydrophobic hexapeptide motif of the mRIP3-RHIM, ‘NSLVAP’, bound a cleft of the HSPA8-SBD binding pocket and received the highest position-specific score (Fig. 5a, b). A leucine (L3) in the middle of the mRIP3 hexapeptide occupied the central deep hydrophobic pocket of HSPA8-SBD (Fig. 5c). The mutation of L3 into negatively charged aspartic acid (L456D) disrupted the binding with HSPA8, indicating that the L3 of the hexapeptide contributes predominantly to HSPA8 recognition (Fig. 5d).

A comparison of RHIM domain sequences revealed that, all RHIM-containing proteins (RIP1, ZBP1, TRIF, and RIP3) harbor similar hexapeptide sequences (marked in yellow) that could potentially be recognized by HSPA8. As illustrated in Fig. 5e, the hexapeptide begins with an uncharged asparagine (N) and includes a hydrophobic central region comprised of leucine (L or M/I, marked in red) as well as one or two additional hydrophobic residues (cyan) that are linked by polar, primarily positively charged residues (X_1_ and X_3_). In 293 T cells, we co-expressed the three initiator RHIM-containing proteins with HSPA8 to test whether HSPA8 recognizes these initiator RHIM-containing proteins as well. As shown in Fig. 5f, all of these RHIM-containing proteins could co-immunoprecipitate with HSPA8.

Following this, we confirmed the critical hydrophobic residues in each initiator RHIM-containing protein that mediate the interaction with HSPA8 using aspartic acid mutational analysis. RIP1’s hydrophobic residues following the RHIM-core, including M547 in the same position as L3 in the mRIP3 hexapeptide, as well as A543 and I549, strongly contribute to the interaction with HSPA8 (Fig. 5g). Mutation of I214 in the first RHIM motif or M272 in the second RHIM motif of ZBP1, both in the same position as L3 in the mRIP3 hexapeptide, reduced the interaction with HSPA8 (Fig. 5h). Furthermore, mutations of TRIF M695, which is also located in the same position as L3 in the mRIP3 hexapeptide, completely disrupted the interaction with HSPA8 (Fig. 5i). Our findings support the notion that a hexapeptide N(X_1_)φ(X_3_) (highlighted in yellow in Fig. 5e) functions as a particular substrate for HSPA8 recognition, with the critical hydrophobic site (symbolized as ‘φ’; φ = L, I, M; highlighted in red in Fig. 5e) stabilizing the binding to HSPA8 by occupying the deep binding pocket.

### HSPA8 inhibits necroptosis initiation by blocking the initiator RHIM-containing protein activation

Necroptosis initiation depends on the activation of the initiator RHIM-containing proteins RIP1, ZBP1, and TRIF. We tested whether HSPA8 affects necroptosis signaling that was activated by different initiator RHIM-containing proteins.

**Fig. 6:**
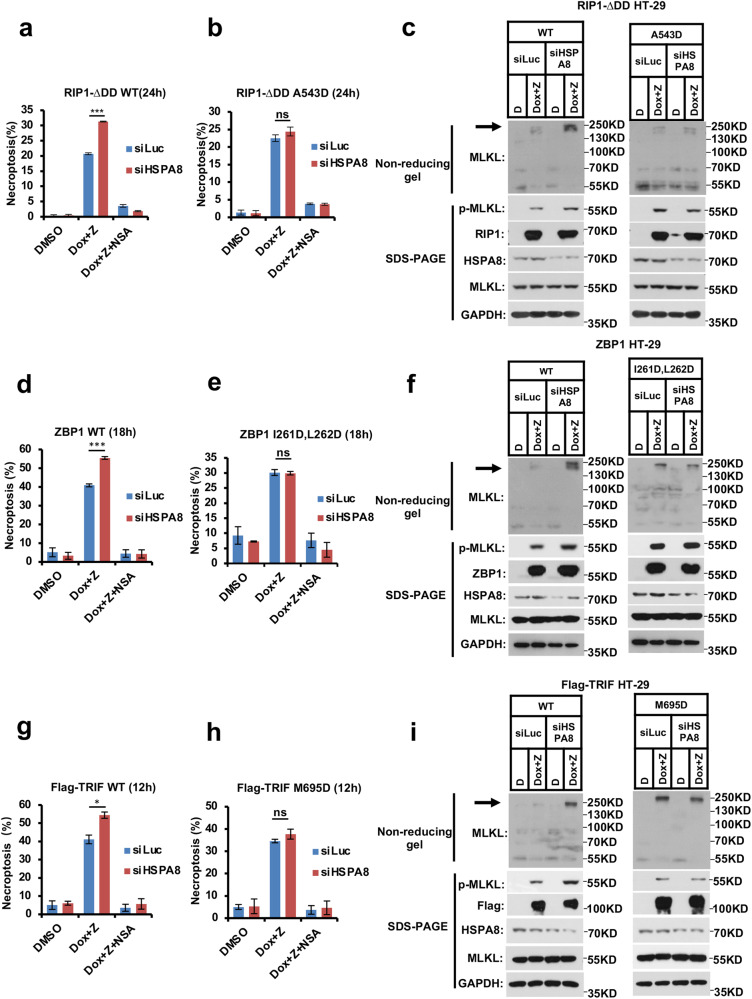
HSPA8 inhibits necroptosis initiation by blocking the initiator RHIM-containing protein activation. **a,** **d,** **g** Knocking down HSPA8 promoted RIP1-, ZBP1-, or TRIF-initiated necroptosis. Necroptosis was initiated by Dox-induced RIP1-∆DD, ZBP1, or TRIF overexpression in the RIP1-KO, WT, or TRIF-KO HT-29 cells, respectively (**a** for RIP1-∆DD; **d** for ZBP1; **g** for TRIF). The cells were transfected with HSPA8 siRNA oligos for 36 h, and then necroptosis was induced by Dox (0.4 μg/mL) plus z-VAD (20 μM) for the indicated time. Cell viability was determined by measuring intracellular ATP levels. The data are represented as mean ± SD of duplicate wells. **b,** **e,** **h** HSPA8 could not block the RHIM-hydrophobic residue mutant RIP1/ZBP1/TRIF-initiated necroptosis. Necroptosis was induced as indicated above (**b** for RIP1-∆DD; **e** for ZBP1; **h** for TRIF). The cells were transfected with HSPA8 siRNA oligos for 36 h, and then necroptosis was induced by Dox (0.4 μg/mL) plus z-VAD (20 μM) for the indicated time. Cell viability was determined by measuring intracellular ATP levels as described in Materials and methods. The data are represented as mean ± SD of duplicate wells. **c,** **f,** **i** HSPA8 could not inhibit the RHIM-hydrophobic residue mutant RIP1/ZBP1/TRIF-directed necroptosis signaling to MLKL. The cells were transfected with HSPA8 siRNA oligos for 36 h, and then necroptosis was induced by adding Dox (0.4 μg/mL) plus z-VAD (20 μM). The whole-cell lysates were subjected to non-reducing gel electrophoresis for analyzing MLKL oligomerization and immunoblotting for detecting phosphorylated MLKL. **c** for RIP1-∆DD; **f** for ZBP1; **i** for TRIF. *P* values were determined by unpaired two-tailed Student’s *t*-test with Welch’s correction. ns, no significance. *P < 0.05; **P < 0.01; ***P < 0.005. All results are reported from one representative experiment from at least three independent repeats.

To explicitly activate RIP1-initiated necroptosis, we used doxycycline (Dox) to induce overexpression of a truncated form of RIP1 (RIP1-∆DD, with the deletion of the Death Domain which is involved in apoptosis activation via binding FADD) in RIP1-knockout HT-29 cells. Necroptosis was induced by overexpressing the WT RIP1-∆DD in combination with the pan-caspase inhibitor z-VAD. Necroptosis was accelerated by silencing HSPA8 or prevented by the MLKL inhibitor NSA (Fig. 6a). Although mutation of the hydrophobic A543 of RIP1-∆DD disrupted the interaction with HSPA8 (as shown in Fig. 5g), it did not affect RIP1-initiated necroptosis. However, knocking down HSPA8 could not further increase RIP1-∆DD-A543D mutant-initiated necroptosis, indicating that the A543D mutation of RIP1 escaped the inhibitory regulation by HSPA8 (Fig. 6b). Consistently, the activation of the necroptosis downstream effector MLKL (the phosphorylation and oligomerization of MLKL) could not be further enhanced by silencing HSPA8 in RIP1-∆DD-A543D-initiated necroptosis (Fig. 6c).

Similarly, in Dox-inducible WT ZBP1- or TRIF-overexpressing HT-29 cells, silencing HSPA8 expression could increase necroptosis (Fig. 6d, g). Mutations of the hydrophobic residues of the RHIM domain (M695D mutation in TRIF; I261D, L262D double mutations in ZBP1) disrupted the interaction with HSPA8 (Fig. 5i; Supplementary information, Fig. S7). Although such mutations did not affect necroptosis, they escaped the negative regulation by HSPA8 (Fig. 6d–i).

### HSPA8 restrains the initiator RHIM-fibril formation

**Fig. 7:**
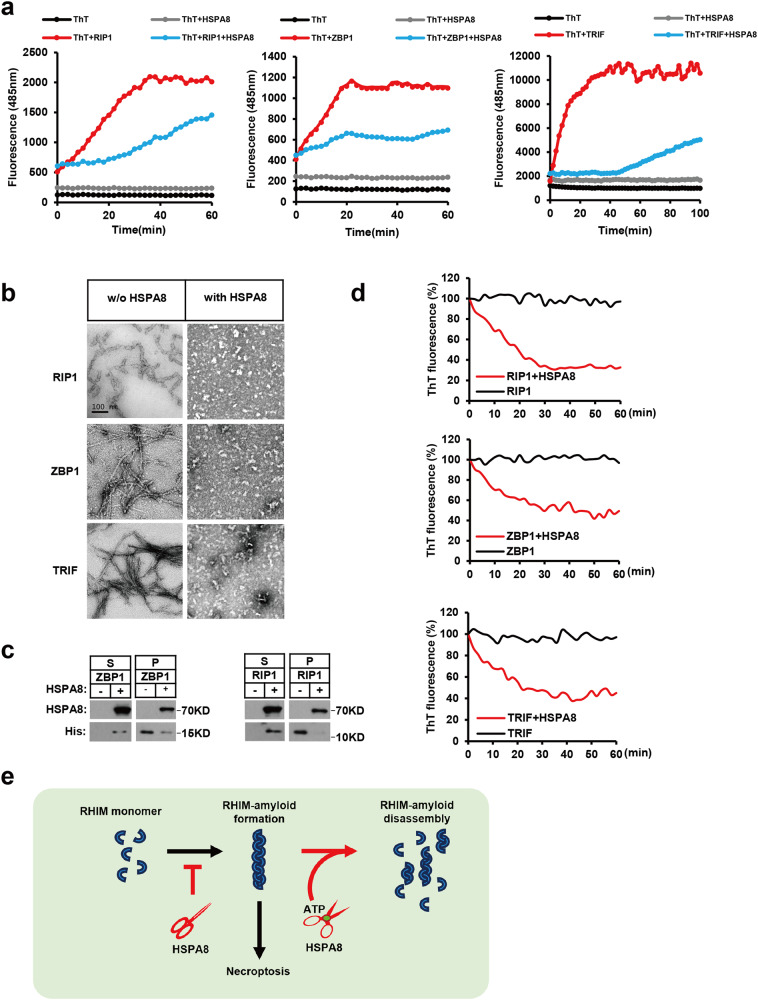
HSPA8 restrains initiator RHIM-fibril formation. **a** The RHIM-based fibril growth was monitored by the ThT staining. RIP1_(497–582)_, ZBP1_(150–293)_ and TRIF_(677–698)_ amyloid assembled in the presence or absence of HSPA8. The newly formed fibrils were stained with ThT (50 μM). The fluorescence intensity was measured every 2 min during the fibril growth process. Excitation wavelength: 430 nm; emission wavelength: 485 nm. **b** Representative negative stain EM showing that HSPA8 disassembled the pre-formed RHIM-fibrils. The pre-formed RHIM-based fibrils were incubated with or without HSPA8 (5 μM) and ATP (4 mM) for 2 h and analyzed by negative stain EM. Scale bar, 100 nm. **c** Immunoblotting analysis of the RHIM-containing proteins disassembled by HSPA8. After the disassembly reaction, the disassembled and the insoluble fibrils were separated by centrifugation and analyzed by immunoblotting with the indicated antibodies. **d** The RHIM-fibril disassembly process was monitored by ThT staining assay. The disassembled fibrils were stained with ThT (50 μM). The fluorescence intensity was measured every 2 min during the disassembly process. ThT signals of sonicated fibrils were subtracted from ThT time course data as baseline signals. Excitation wavelength: 430 nm; emission wavelength: 485 nm. **e** The hybrid working model for HSPA8 reversing functional RHIM-based amyloids. HSPA8 directly targets the RHIM-containing proteins and prevents the RHIM-amyloid formation; HSPA8 disassembles the pre-formed RHIM-amyloids coupled with ATP hydrolysis. All results are reported from one representative experiment from at least three independent repeats.

Following this, we investigated the effect of HSPA8 on the fibrillation of RIP1, ZBP1, and TRIF in vitro. The pre-formed RHIM-fibrils of these proteins were sonicated into ‘seeds’ and then incubated with the corresponding monomeric proteins. The fibril growth of these three initiator RHIM-containing proteins was suppressed by HSPA8 in the fibrillation system; in the absence of HSPA8, the three RHIM-based fibrils grew rapidly and reached a plateau within 40 min (Fig. 7a).

We also investigated HSPA8-mediated disassembly of pre-formed RIP1-, ZBP1-, and TRIF-fibrils. When pre-formed RHIM-based fibrils were incubated with HSPA8, all of the fibrils disassembled and fragmented into small globular oligomer structures; in the absence of HSPA8, those fibrils maintained their filamentous morphology as detected by EM (Fig. 7b). The disassembled fibrils were resolved into the supernatant (S), separated from the fibrils remaining in the pellet (P) by centrifugation, which was confirmed by immunoblotting (Fig. 7c). HSPA8 disassembled all RHIM-fibrils within 1 h, according to ThT staining (Fig. 7d).

Consistently, the amyloids formed by full-length RHIM-containing proteins could also be disassembled by HSPA8. Overexpression of RHIM-containing proteins resulted in the formation of self-oligomers in the cellular context of 293T cells. The intermolecular interaction between RHIM-containing proteins was stably preserved when RHIM-oligomers were isolated from whole-cell lysate by immunoprecipitation; however, when we incubated them with recombinant HSPA8, the RHIM-oligomers were disassembled (Supplementary information, Fig. S8a–e). Furthermore, after HSPA8 was silenced, the amyloidal signal of the RIP3-containing necrosome was significantly enhanced, as detected by ThT staining (Supplementary information, Fig. S8f).

## Discussion

Similar to how helicases use the energy from ATP hydrolysis to catalyze the separation of duplex nucleic acids, we defined a new type of enzyme, amyloidase, that catalyzes the disassembly/separation of fibrous functional amyloids into non-functional monomers. Arising findings revealed that functional amyloids participate in a variety of cellular processes, such as melanosome biogenesis and the storage of peptide hormones in pituitary secretory granules.^1,2,4^ Our current study identified HSPA8 as the first amyloidase in mammalian cells, with the specific role of reversing RHIM-amyloids to prevent necroptosis. Given that different functional amyloids modulate different signaling pathways, we predict that their corresponding amyloidase(s) may constitute a highly versatile family, each recognizing a different (class of) functional amyloid(s) to protect cells from overwhelmed signal amplification. In the future, different signaling pathway-based amyloidase screenings are expected to expand the amyloidase family.

Reversibility should be a key feature shared by all functional amyloids. Previously, a delicate study reported the reversal mechanism of Pmel17 amyloid by environmental pH changes during melanosome biogenesis: when the melanosome is immature, Pmel17 aggregates to form amyloids under the acidic conditions; after melanosome matures, the Pmel17 fibrils rapidly dissolve to generate benign monomers in the neutral environment.^44,45^ Unlike the melanosomal amyloid formation regulated by intramelanosomal pH changes, the cytoplasmic functional amyloids including RHIM-amyloids are highly buffered from pH fluctuations. Even in the cell-free system, pH changes had little effect on the ultrastability of the recombinant RHIM fibrils. We speculate that the reversibility of most cytoplasmic functional amyloids must be catalyzed by amyloidase(s).

The rapidity of HSPA8 alone disassembling RHIM-amyloid was unexpected. HSPA8 alone was unable to disaggregate other protein amyloids without the sophisticated co-chaperone system, such as thermally denatured luciferase or unfolded Parkinson’s disease-linked presynaptic protein α-syn fibrils, even after 30 days’ co-incubation.^39,40^ An in vitro study found that a co-chaperone system of HSPA8-J-protein-HSPH2 (also known as HSP70-J-protein-HSP110 system) could synergistically disaggregate the above-mentioned misfolded proteins.^46–48^ It is thus obvious that HSPA8-mediated direct regulation of functional RHIM-amyloids is hypostatic in contrast to the co-chaperone system that regulates cytotoxic protein aggregates, which implies that HSPA8 recognizes functional RHIM-based amyloid as its direct legitimate substrate for disassembly in vivo to maintain homeostasis.

In contrast to the majority of HSP70 family members, such as HSPA1A and HSPA1B, which are stress-inducible, HSPA8 is constitutively expressed in cells.^49,50^ The turnover of RHIM-containing proteins was previously reported to be regulated by autophagy over a long period of time,^51^ whereas our current discovery reveals that constitutively expressed HSPA8 acts as an amyloidase that suppresses necroptosis in a timely manner.

Recent studies have reported the involvement of HSP70 family proteins in the regulation of necroptosis. For instance, Srinivasan et al.^52^ demonstrated that an Hsp70 inhibitor, JG-98, led to RIP1-dependent cell death. JG-98 targets multiple HSP70 members, including HSPA8, which is consistent with our findings suggesting the involvement of HSPA8 in necroptosis regulation. Furthermore, a different HSP70 member, HSP70 (HSPA1A), was reported to promote MLKL polymerization and necroptosis. Targeting HSPA1A was shown to inhibit MLKL polymerization and block necroptosis.^53,54^ In addition to the HSP70s, a cytosolic heat shock protein 90 (HSP90) and its co-chaperone CDC37 have been shown to play a crucial role in RIP3 activation during necroptosis.^55^ This is likely attributed to the facilitation of RIP kinase folding by the CDC37–HSP90 complex.^56^ HSP90 was also found to regulate the expression and activity of RIP1/RIP3/MLKL under compression in nucleus pulposus-derived stem/progenitor cells (NPSCs).^57^ Additionally, HSP90 has been found to modulate the stability of MLKL and is required for TNF-induced necroptosis.^58^ It has also been demonstrated that HSP90 activity is necessary for MLKL oligomerization, membrane translocation, and the induction of necroptotic cell death.^59^ Taken together, these findings indicate that different members of the HSP family play distinct roles in the necroptosis pathway.

HSPA8 recognizes a consensus hydrophobic peptide motif of the RHIM region. Normally, the hydrophobic sequence stretches are buried in the core of the correctly folded proteins, and they only become exposed when the protein is denatured. It is unclear how the hydrophobic peptide motif of RHIM-containing proteins is accessible for HSPA8 binding under normal conditions. In addition, the hydrophobic amino acids in front of the RHIM core, like ZBP1 I261/L262 (as shown in Fig. 6d–f and Supplementary information, Fig. S6), were also necessary for HSPA8 binding discovered by our mutational analysis. Further structural research is required to understand how these hydrophobic amino acids outside of the hexapeptide motif support binding to HSPA8.

HSPA8 is comprised of an N-terminal NBD and a C-terminal SBD, which are linked by a short inter-domain linker. NBD is also critical for regulating peptide binding and releasing, depending on different ATP-binding status. In the ATP-bound state, the SBD domain-mediated substrate-binding affinity is low, and the lid of SBD is open; after substrate-binding, ATP is hydrolyzed to ADP, the lid of SBD closes, and the substrate binding affinity increases. Reversely, SBD also affects the ATPase rate of NBD by binding with substrate. The linker between NBD and SBD participates in inter-domain communication.^35^ Full details of the allosteric coupling of SBD with NBD, specifically how the flexibility of the linker domain contributes to regulation of RHIM binding, and how the RHIM-hexapeptide binding with SBD influences the ATPase rate of NBD, require further investigation and will be established through detailed structural analysis.

RHIM-containing proteins are highly conserved across species and play various roles, including anti-bacterial innate immunity response in insects and the non-self-recognition process (termed ‘heterokaryon incompatibility’) in filamentous fungi.^60–63^ The mechanism of HSPA8 restraining RHIM-amyloid-mediated signal transduction will inspire forthcoming studies to test whether it is applicable in a wide range of physiological scenarios across different species, including but not limited to necroptosis.

## Materials and methods

### Cell culture

L929, HeLa, 3T3, HT-29, HeLa-RIP3, hMLKL_(1–178)_-2×FKBP-293FT, mRIP3-2×FKBP-3T3, and 293FT cells were cultured in Dulbecco’s modified Eagle’s medium (DMEM, Corning, Cat# 10-013-CV) supplemented with 10% fetal bovine serum (FBS, GIBCO, Cat# 10099141) and 100 units/mL penicillin/streptomycin. All cells were cultured at 37 °C in a 5% CO_2_ incubator. All cell lines were tested to be mycoplasma-negative by the standard PCR method.

### Plasmids

EGFP (non-tagged), HSPA8 (mouse and human), siRNA-resistant HSPA8 (mouse and human), hRIP3, and mRIP3-2×FKBP were cloned into pHAGE plasmid and delivered into cells with lentivirus. The pHAGE plasmid was kindly provided by Dr. Weiguo Zou in SIBCB. RIP1-ΔDD, ZBP1, and TRIF were cloned into pLVX plasmid for Tet-on Dox-induced protein expression. The pLVX plasmid was kindly provided by Dr. Daming Gao in SIBCB. For transient expression in 293FT or HeLa cells, cDNAs were cloned into pLIBIN plasmid, which was kindly provided by Dr. Xiaodong Wang in NIBS. For expression of the recombinant proteins in *E. coli* system, hHSPA8, hHSPA8-K71A, hHSPA8-CT, and hRIP3_(388–518)_ tagged with 6×His-Sumo were subcloned into the pE-SUMO vector. Point mutations were introduced by site-directed PCR strategy.

### Antibodies and reagents

For immunoblotting, antibodies were purchased from Sigma-Aldrich (HRP-anti-Flag, Cat# A8592; anti-Myc-HRP, Cat# 16-213), Cell Signaling Technology (HRP-anti-rabbit IgG, Cat# 7074P2; HRP-anti-mouse IgG, Cat# 7076; anti-β-actin, Cat# 4967; anti-RIP1, Cat# 3493; anti-capsase8, Cat# 9746), ABclonal (anti-GAPDH, Cat# AC033; anti-HSPA8, Cat# A10898), Prosci (anti-mouse RIP3, Cat# 2283) and Abcam (anti-LAMP2A, ab125068; anti-V5-HRP, ab1325; anti-6×His, ab18184; anti-human p-MLKL, ab187091; anti-mouse p-MLKL, ab196436). Anti-human MLKL, anti-mouse MLKL and anti-human RIP3 antibodies are generated by our own laboratory.

Recombinant TNF was purified in the lab. ATP (#A600020-0005) was purchased from Sangon. Smac mimetics LCL161 (HY-15518), GSK872 (HY-101872), and NSA (HY-100573) were purchased from MedChemExpress. Trypsin (#25200072) was purchased from ThermoFisher Scientific. ThT (#596200), Flag beads (#M8823), Myc beads (#E6654), V5 beads (#A7345), Nec-1 (#N9037) phosphoenolpyruvate (#P7127) and pyruvate kinase (#P7768) were purchased from Sigma-Aldrich. Nec-1s (#2263) was purchased from BioVision. The TRIF_(677–698)_ proteins and Z-VAD-FMK were custom-made by WuXi AppTec. Recombinant RHIM-containing proteins (RIP1_(498–582)_, RIP3_(418–518)_, and ZBP1_(150–293)_) and recombinant α-syn were generated previously.

### Recombinant protein purification

Plasmids were transfected into *E. coli* BL21 (DE3) competent cells, and cultured at 37 °C. Protein expression was induced by adding 1 mM IPTG (AMRESCO, Cat# 0487) at OD_600_ = 0.6–0.8. Cells were cultured at 16 °C overnight. The harvested cells were lysed by lysis buffer containing 150 mM NaCl (for hHSPA8, hHSPA8K71A, and hHSPA8-CT) or 300 mM NaCl (for RIP3_(388–518)_ in Fig. 4), 50 mM Tris-HCl, pH 8.0, 200 μg/mL PMSF and 0.2 mM β-mercaptoethanol, and sonicated. The supernatant was incubated with Ni-NTA agarose at 4 °C for 1 h, respectively. The Ni-NTA agarose was subsequently washed and then eluted with 500 mM imidazole. The eluted protein was further purified by gel filtration chromatography using the Superdex 200 10/300 GL column (GE Healthcare) at 4 °C in an ÄKTA FPLC system (GE Healthcare). Protein concentrations were determined by Bradford assay using bovine serum albumin (BSA) as standard.

### siRNA screening

siRNA screening was performed using siRNA pools covering most of the mouse genome (mouse kinase and phosphatase, G protein-coupled receptor, protease, ion channels, ubiquitin conjugation, druggable genome and remaining genome libraries, Dharmacon). Mouse fibrosarcoma cell line L929 (WT and MLKL KO) cells cultured in 384-well white plates (Corning) were transfected with 50 nM siRNA by reverse transfection method using Lipofectamine™ RNAiMAX Transfection Reagent according to a protocol from the manufacturer (ThermoFisher Scientific). 72 h after the transfection, cell viability was determined by measuring luminescence-based ATP levels using CellTiterGlo ATP assay (Promega). The positive control (siCaspase8) and negative control (siLuciferase) were run in every plate.

### siRNA sequences

mouse HSPA8: CAAGAGAGCUGUCCGCCGU, AGUCACAGAUCCAUGAUAU

human HSPA8: GAACAAGAGAGCUGUAAGA, GGACGCAGAUUUGAUGAUG

human LAMP2A: CUCAAUAGCAGCACCAUUA, GCAUGUAUUUGGUUAAUGG

mouse LAMP2A: GAUCAACACCUUUAACCUA, CAAGGAAGCAUCUCAUUAU

human MLKL: CAAACUUCCUGGUAACUCA

human Caspase8: GATCAGAATTGAGGTCTTT

### ATP assay

Cell viabilities (the intracellular ATP levels of the remaining cells) were measured with the CellTiter-Glo Luminescent Cell Viability Assay Kit (Promega) according to the manufacturer’s instructions. Necroptosis or apoptosis was quantified by subtracting the cell viability from the initial total cell viability.

### SYTOX Green staining assay

SYTOX Green (Invitrogen, 100 nM) was added to the cell culture medium to trace the plasma membrane breakdown at 10 min before microscopic imaging. The intranuclear DNA fluorescent signal of SYTOX Green indicated the membrane leakage. The SYTOX Green^+^ (necroptotic) cells were quantified by IncuCyte.

### Non-reducing SDS-PAGE analysis

The whole-cell lysate was mixed with SDS sample buffer without β-mercaptoethanol and boiled at 100 °C for 5 min. The prepared samples were subjected to SDS-PAGE, followed by immunoblotting.

### His pull-down assay

GST-tagged HSPA8-SBD_(385–647)_ domain and His-tagged RIP3-RHIM region with the mutation of the RHIM-core (His-Sumo-RIP3_(388–518)_-4A) were purified from *E. coli* separately and mixed together at the ratio of 1:1 (50 μM each) for 6 h at 4 °C. The His-tagged RIP3-RHIM was used as bait; the GST-tagged HSAP8 was used as prey and was pulled down by His-tagged RIP3-RHIM.

### EM analysis

One drop of fibril solution (~15 μL) was deposited onto a 200-mesh carbon-coated grid. The grid was washed with Milli-Q water and then stained with 2% uranyl acetate for 1 min. The excess liquid was removed by Kim Wipe paper and the grid was air dried. The electron micrographs were recorded with an electron microscope FEI Tecnai Spirit (120 kV).

### Negative staining immunogold EM

The grid was treated with EM blocking medium (1% FBS, 1% BSA, 0.1% Tween-20 in PBS) for 30 min to prevent non-specific binding of antibodies to the grid surface. Subsequently, the grid was incubated with anti-HSPA8 antibody (diluted 1/100) for 1 h. Afterward, the grid was washed with PBS and incubated with secondary gold probes for 1 h. Following another wash with PBS, the grid was rinsed with Milli-Q water and then stained with 2% uranyl acetate for 1 min. Excess liquid was carefully removed using Kim Wipe paper, and the grid was left to air dry. Electron micrographs were captured using an electron microscope, specifically the FEI Tecnai Spirit (120 kV).

### Fibril growth assay

The pre-formed RHIM-containing protein fibrils were prepared by incubating the freshly purified RHIM-containing proteins at 37 °C for 48 h. Then the fibrils were sonicated into ‘seeds’, with 8% vibration amplitude, 2 s on/2 s off for 20 cycles on ice. Fibril growth was started by incubating the seeds (0.5 μM) with the monomer RHIM-containing protein peptides (5 μM). The fibril growth assays were performed in the presence or absence of HSPA8 at 30 °C for the indicated times. The insoluble fibrils were fractionated by centrifugation (12,000× *g*, 10 min) and analyzed by EM.

### Structural modeling recognition mode of RIP peptides by HSP8A-SBD

Models of HSP8A SBD bound with RHIM peptides were generated using a local version of AlphaFold-Multimer, installed using the open-source code and instructions available at https://github.com/deepmind/alphafold.

### Fibril disassembly assay

The pre-formed fibrils (5 μM) were incubated with or without HSPA8 (5 μM) at 30 °C for 120 min in the disassembly buffer (50 mM HEPES-KOH, pH 7.5, 50 mM KCl, 5 mM MgCl_2_, 4 mM ATP). ATP regeneration system (3 mM phosphoenolpyruvate, 20 ng/mL pyruvate kinase) and DTT (2 mM) were also added.

### Full-length RHIM-containing protein disassembly assay

Myc-RIP1 and Flag-RIP1, Myc-RIP3 and Flag-RIP3, HA-ZBP1 and Flag-ZBP1, and Myc-TRIF and Flag-TRIF were respectively transfected into 293FT for 24 h. The harvested cells were lysed with 1% Triton X-100 buffer. The whole-cell lysate was incubated with Myc- or HA-beads for coimmunoprecipitation. The beads were washed three times with protein buffer (50 mM Tris, 150 mM NaCl, pH 8.0). Aliquots of the beads were incubated with 0 μg, 0.5 μg, or 1 μg HSPA8 for 2 h at 30 °C. The disaggregated RHIM-containing proteins were separated from pellets and retained in supernatant by centrifugation (5000× *g*, 5 min). The protein components of each fraction were analyzed by immunoblotting.

### SIRS model

C57BL/6 J mice were purchased from Shanghai Lingchang Biotechnology Co., Ltd. *Rip3*^*−/−*^ mice were produced by targeted gene deletion. Mice were maintained in a pathogen-free environment. Mice of ~8 weeks old were given intravenous injections of TNFα (7 μg). Vehicle control (5% DMSO + 45% PEG 300 + 5% Tween 80) or HSPA8 inhibitor (PES, 45 mg/kg, dissolved in the vehicle control solution) was injected at 30 min before TNFα injection. Mouse survival was monitored at 120-min intervals. The body temperature was monitored every 2 h.

After mice were euthanized, small intestines were collected immediately and washed with PBS buffer. Then the small intestines were rolled in the shape of Swiss roll and fixed in 4% formalin buffer for 48 h. The fixed tissues were dehydrated in ethanol, cleared in xylene, and embedded in paraffin. 5-μm thick small intestine sections were cut and stained with hematoxylin and eosin (H&E).

### Mouse model of HSPA8 inhibitor-induced toxicity

#### Hypothermia analysis

The WT, *Rip3*^*−/−*^, and *Mlkl*^*−/−*^mice were injected with HSPA8 inhibitor AZ (45 mg/kg) or PES (90 mg/kg). The body temperature was measured at 120-min intervals.

#### Intestine toxicity analysis

The WT, *Rip3*^*−/−*^, and *Mlkl*^*−/−*^ mice were treated with the HSPA8 inhibitor PES (90 mg/kg) once daily for two consecutive days. Afterward, the intestines were collected and fixed in 4% paraformaldehyde at 4 °C for 48 h. Paraffin sections were prepared following standard protocols, and hematoxylin was used for counterstaining.

### Supplementary information

Supplementary information, Fig. S1

Supplementary information, Fig. S2

Supplementary information, Fig. S3

Supplementary information, Fig. S4

Supplementary information, Fig. S5

Supplementary information, Fig. S6

Supplementary information, Fig. S7

Supplementary information, Fig. S8

Supplementary information, Table S1

## Data Availability

The original data used to support the findings of this study are available from the corresponding author upon request.
