# Supplementary material for: HSPA8 acts as an amyloidase to suppress necroptosis by inhibiting and reversing functional amyloid formation
Source: Cell Res. 2023 Aug 14;33(11):851–66. doi: 10.1038/s41422-023-00859-3 (PMC10624691; doi:10.1038/s41422-023-00859-3)
Supplement: Supplementary file 1 — Supplementary information, Fig. S1 [file 41422_2023_859_MOESM1_ESM.pdf]

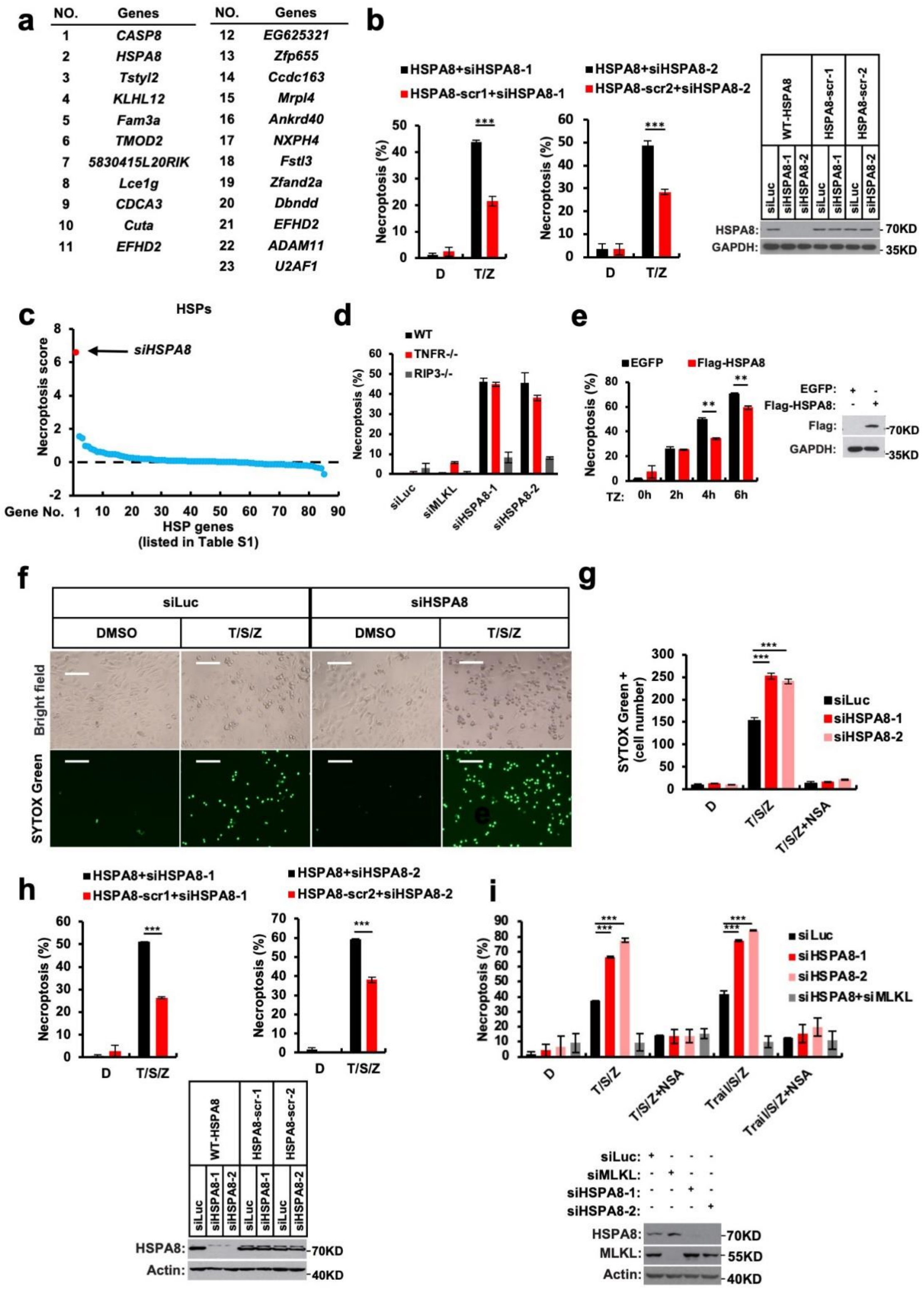

Supplementary information, Fig. S1 HSPA8 inhibits necroptosis.

a List of the top genes from the siRNA screening (as described in Fig. 1a). These top hits were further reconfirmed by shRNA knockdown assay in L929 cells.

b Reintroducing the siRNA-resistant HSPA8 expression in HSPA8-knocked-down L929 cells rescued the necroptosis-inhibitory role of HSPA8. The siRNA-resistant mouse HSPA8 cDNAs were delivered into the L929 cells by lentivirus infection. Then the cells were transfected with the corresponding siHSPA8 oligos. Thirty-six hours later, necroptosis was induced by treating cells with T/Z for 3 hours. Cell viability was determined by measuring intracellular ATP levels**.** The data are represented as the mean ± SD of duplicate wells. The HSPA8 knockdown and rescue efficiencies were tested by immunoblotting (right panel).

c The necroptosis-inhibitory score of the heat shock protein family genes. The necroptosis-inhibitory score= (ATP level of MLKL-KO L929)/(ATP level of L929)-100%.

d Knocking out TNFR1 did not block siHSPA8-induced necroptosis in L929 cells. Cells were transfected with indicated siRNA oligos. Seventy-two hours later, spontaneous necroptosis was measured by ATP level. Cell viability was determined by measuring intracellular ATP levels as described in Methods. The data are represented as the mean ± SD of duplicate wells.

e Overexpression of HSPA8 inhibited necroptosis. The cDNAs encoding Flag-tagged HSPA8 were introduced into L929 cells through lentiviral infection. Necroptosis was induced by treating the cells with T/Z for the specified durations. Cell viability was determined by measuring intracellular ATP levels. The data are represented as the mean ± SD of duplicate wells.

f Knocking down HSPA8 promoted necroptotic cytolysis. The expression of Flag-tagged RIP3 was induced by adding 0.4 μg/ml doxycycline (dox) for 12 hours. Then the RIP3-expressing HeLa cells (HeLa-RIP3) were transfected with indicated siRNAs. Thirty-six hours later, necroptosis was induced by T/S/Z for 10 hours. Plasma membrane breakdown was traced with SYTOX Green (100 nM) staining. Scale bar: 200 μm. Necroptosis inducer T/S/Z: T, TNF-α (20 ng/ml); S, Smac mimetic (100 nM); Z, z-VAD (20 μM). Necroptosis inhibitor, NSA: Necrosulfonamide (1 μM).

g Quantification of the cytolytic (necroptotic) HeLa-RIP3 cells as shown in **f** by IncuCyte. The data are represented as the mean ± SD of triplicate SYTOX Green+ lytic cell numbers from three different views for each condition.

h Reintroducing the siRNA-resistant HSPA8 expression in HSPA8-knocked-down HeLa-RIP3 cells rescued the necroptosis-inhibitory role of HSPA8. The siRNA-resistant human HSPA8 cDNAs were delivered into the HeLa-RIP3 cells by lentivirus infection. Then cells were transfected with the corresponding siHSPA8 oligos. Thirty-six hours later, necroptosis was induced by treating cells with T/S/Z for 10 hours. Cell viability was determined by measuring intracellular ATP levels as described in Methods. The data are represented as the mean ± SD of duplicate wells. The HSPA8 knockdown and rescue efficiencies were tested by immunoblotting (bottom panel).

i HSPA8 inhibited either TNF- or TRAIL-induced necroptosis. Necroptosis was induced in HeLa-RIP3 cells by treating cells with T/S/Z or TRAIL/S/Z. The siHSPA8-enhanced necroptosis was blocked by treating cells with MLKL inhibitor NSA or knocking down MLKL expression. Cell viability was determined by measuring intracellular ATP levels. Necroptosis inducer TRAIL/S/Z: TRAIL (50 ng/mL); S, Smac mimetic (100 nM); Z, z-VAD (20 μM). The data are represented as the mean ± SD of duplicate wells. The HSPA8 and MLKL knockdown efficiencies were tested by immunoblotting (bottom panel).

p values were determined by unpaired two-tailed Student's t-test with Welch's correction. $^{**}p < 0.01$; $^{***}p < 0.005$. All results are reported from one representative experiment from at least three independent repeats.
