# Supplementary material for: HSPA8 acts as an amyloidase to suppress necroptosis by inhibiting and reversing functional amyloid formation
Source: Cell Res. 2023 Aug 14;33(11):851–66. doi: 10.1038/s41422-023-00859-3 (PMC10624691; doi:10.1038/s41422-023-00859-3)
Supplement: Supplementary file 2 — Supplementary information, Fig. S2 [file 41422_2023_859_MOESM2_ESM.pdf]

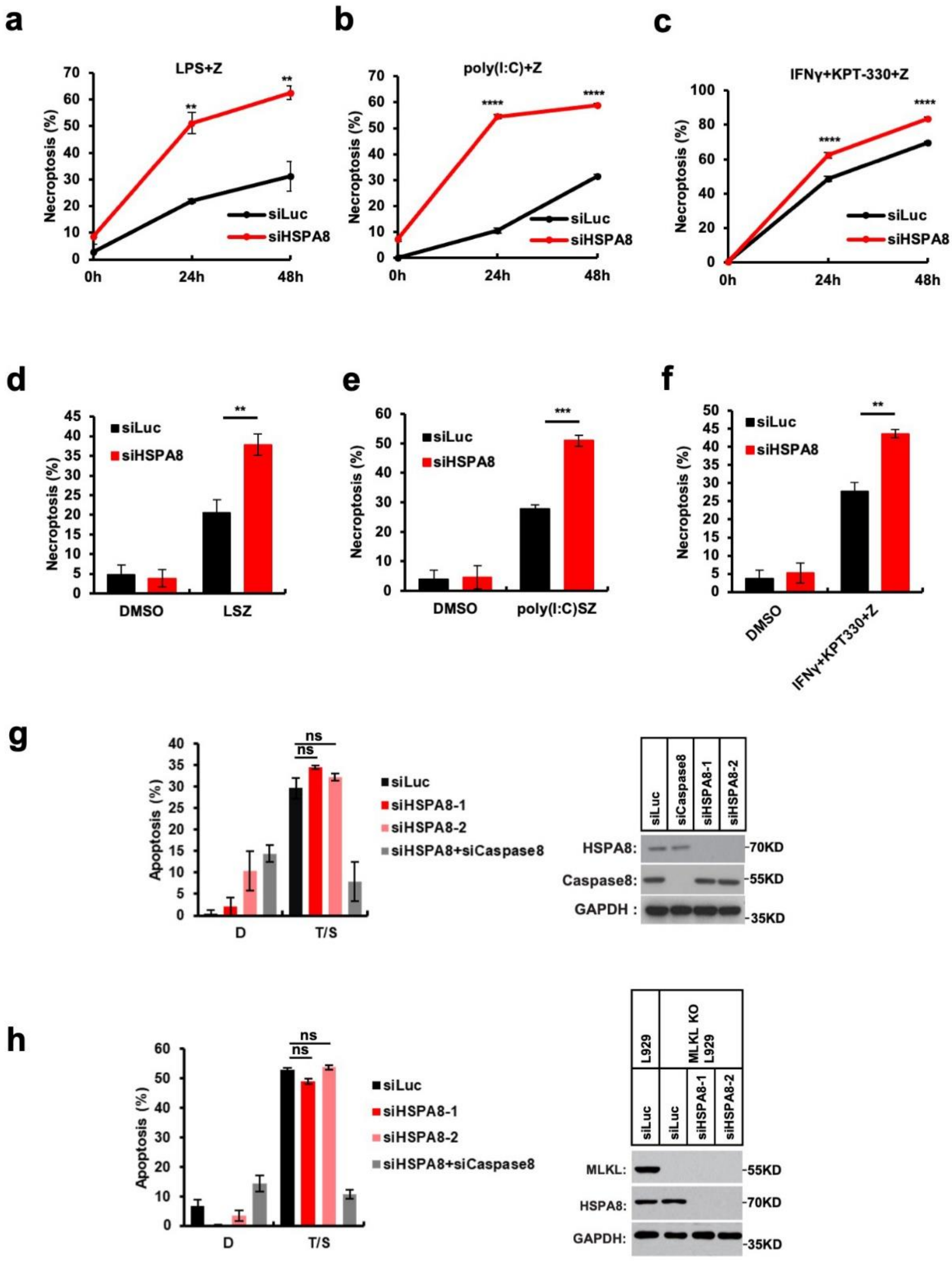

Supplementary information, Fig. S2 HSPA8 inhibits necroptosis induced by multiple stimuli but has no effect on apoptosis.

a-f HSPA8 suppressed TLR3-, TLR4-, and ZBP1-initiated necroptosis. **(a-c)** L929 cells were transfected with indicated siRNAs. Thirty-six hours later, necroptosis was induced by LPS/Z, poly(I: C)/Z, or IFNγ /KPT-330/Z for indicated time. LPS/Z for **(a)**, poly(I: C)/Z for **(b)**, or IFNγ/KPT-330/Z for **(c)**. **(d-f)** HeLa-RIP3 cells were transfected with indicated siRNAs.

Thirty-six hours later, necroptosis was induced by LPS/Smac/Z, poly(I: C)/Smac/Z, or IFNγ /KPT-330/Smac/Z, LPS/Smac/Z for (**d**), ploy(I:C)/Smac/Z for **(e)**, or IFNγ /KPT-330/Z for **(f)**.

Cell viability was determined by measuring intracellular ATP levels. The data are represented as the mean ± SD of duplicate wells.

g Knocking down HSPA8 did not affect apoptosis in human HeLa cells. HeLa cells were transfected with the indicated siRNA oligos. Thirty-six hours later, apoptosis was induced by treating cells with T/S for 6 hours. Cell viability was determined by measuring intracellular ATP levels. Apoptosis inducer T/S: T, TNF-α (20 ng/ml); S, Smac mimetic (100nM). The data are represented as the mean ± SD of duplicate wells. The HSPA8 knockdown efficiency was tested by immunoblotting analysis (right panel).

h Knocking down HSPA8 did not affect apoptosis in mouse L929 cells. The MLKL-KO L929 cells were transfected with the indicated siRNA oligos. Thirty-six hours later, apoptosis was induced by treating cells with T/S for 12 hours. Cell viability was determined by measuring intracellular ATP levels. The data are represented as the mean ± SD of duplicate wells. The HSPA8 knockdown efficiency was shown by immunoblotting analysis (right panel).

p values were determined by unpaired two-tailed Student's t-test with Welch's correction. ns, no significance; $^{**}p < 0.01$; $^{***}p < 0.005$. All results are reported from one representative experiment from at least three independent repeats.
