# Supplementary material for: HSPA8 acts as an amyloidase to suppress necroptosis by inhibiting and reversing functional amyloid formation
Source: Cell Res. 2023 Aug 14;33(11):851–66. doi: 10.1038/s41422-023-00859-3 (PMC10624691; doi:10.1038/s41422-023-00859-3)
Supplement: Supplementary file 3 — Supplementary information, Fig. S3 [file 41422_2023_859_MOESM3_ESM.pdf]

a

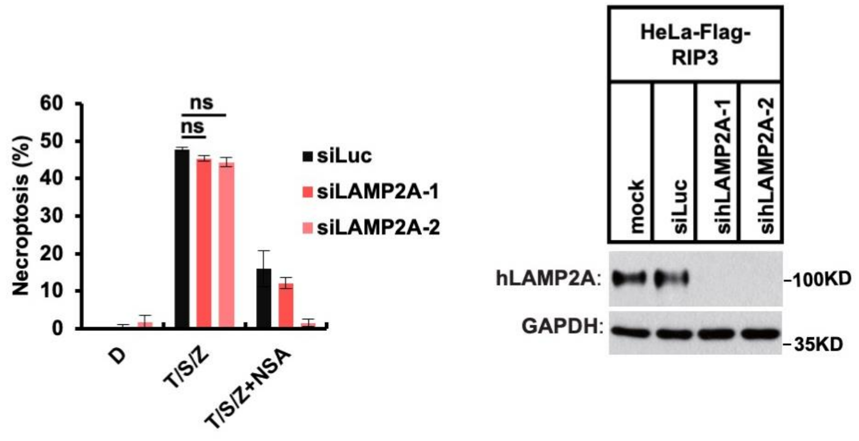

b

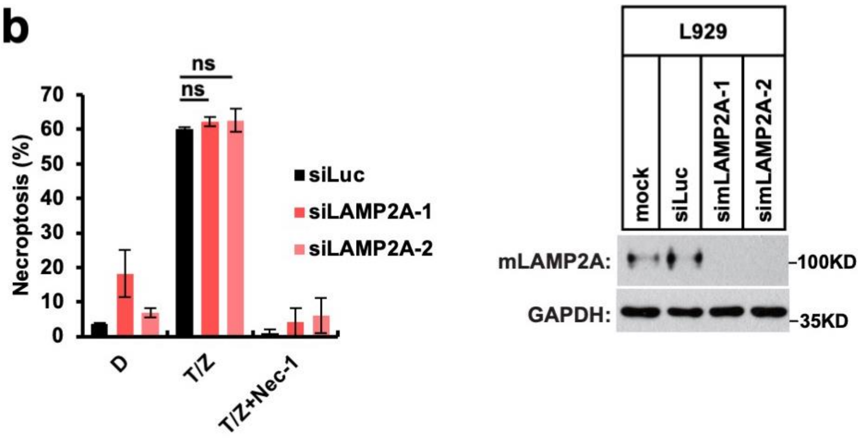

Supplementary information, Fig. S3 HSPA8 blocks necroptosis, which is independent of the CMA machinery.

a Knocking down LAMP2A did not affect necroptosis in human HeLa-RIP3 cells. HeLa-RIP3 cells were transfected with indicated siRNA oligos. Thirty-six hours later, necroptosis was induced by treating cells with T/S/Z for 10 hours. Cell viability was determined by measuring intracellular ATP levels. The data are represented as the mean ± SD of duplicate wells. The LAMP2A knockdown efficiency was tested by immunoblotting analysis (right panel).

b Knocking down LAMP2A did not affect necroptosis in L929 cells. L929 cells were transfected with indicated siRNA oligos. Thirty-six hours later, necroptosis was induced by

treating cells with TZ for 3 hours. Cell viability was determined by measuring intracellular ATP levels. The data are represented as the mean ± SD of duplicate wells. The LAMP2A knockdown efficiency was tested by immunoblotting analysis (right panel).

p values were determined by unpaired two-tailed Student's t-test with Welch's correction. $^{**}p < 0.01$; $^{***}p < 0.005$. All results are reported from one representative experiment from at least three independent repeats.
