# Supplementary material for: HSPA8 acts as an amyloidase to suppress necroptosis by inhibiting and reversing functional amyloid formation
Source: Cell Res. 2023 Aug 14;33(11):851–66. doi: 10.1038/s41422-023-00859-3 (PMC10624691; doi:10.1038/s41422-023-00859-3)
Supplement: Supplementary file 4 — Supplementary information, Fig. S4 [file 41422_2023_859_MOESM4_ESM.pdf]

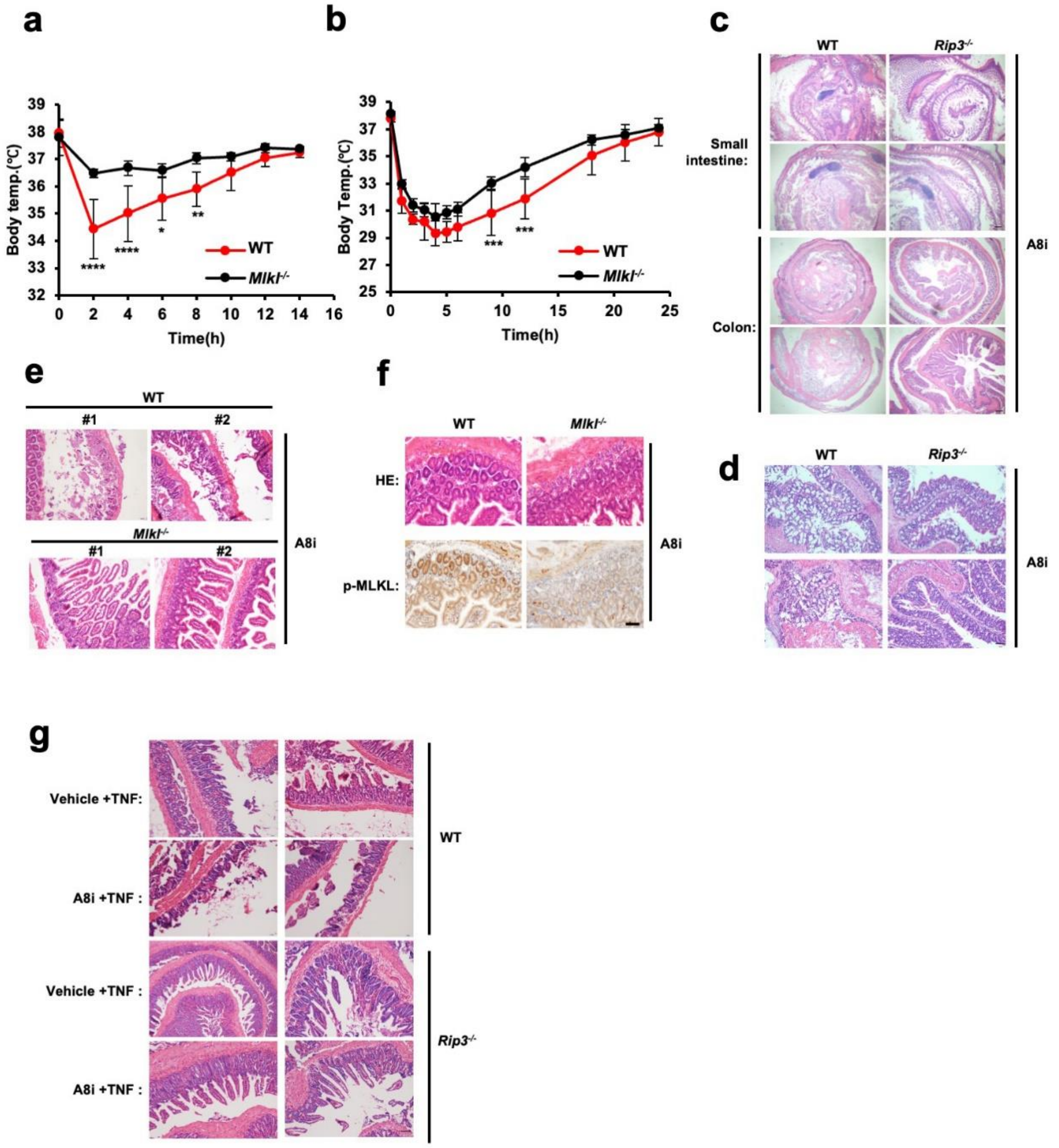

Supplementary information, Fig. S4 HSPA8 inhibition induces necroptosis-mediated toxicity in mice.

a MLKL deficiency prevented Apoptozole (AZ) injection-induced hypothermia in mice. The wild type (WT) and *Mlkl*$^{-/-}$ mice were injected with HSPA8 inhibitor Apoptozole (45 mg/kg). The body temperature was measured at 120min intervals. n=6 mice for each group.

b MLKL deficiency prevented PES injection-induced hypothermia in mice. The wild type (WT) and *Mlkl*$^{-/-}$ mice were injected with HSPA8 inhibitor Pifithrin-μ (90 mg/kg). The body temperature was measured at 120min intervals. n=6 mice for each group.

c Representative histopathology of *Rip3*$^{-/-}$ mice's intestines and colons following injection of

HSPA8 inhibitors.

d A zoomed-in image of colonic histopathology after injection of HSPA8 inhibitor.

e MLKL deficiency prevented PES injection-induced intestine toxicity in mice. Wild-type (WT) and *Mlkl*$^{-/-}$ mice were treated with the HSPA8 inhibitor Pifithrin-μ (90 mg/kg) once daily for two consecutive days. Afterward, the intestines were collected and fixed in 4% paraformaldehyde at 4°C for 48 hours. Paraffin sections were prepared following standard protocols, and hematoxylin was used for counterstaining. Scale bars representing 50 μm were included in the images.

f Phosphorylated MLKL was detected in the intestines of WT mice but not *Mlkl*$^{-/-}$ mice following PES injection. Wild-type (WT) and *Mlkl*$^{-/-}$ mice were treated with the HSPA8 inhibitor Pifithrin-μ (90 mg/kg) once daily for two consecutive days. Afterward, the intestines were collected and fixed in 4% paraformaldehyde at 4°C for 48 hours. Paraffin sections were prepared following standard protocols. Immunohistochemistry was performed using antibodies against murine p-MLKL. Sections were counterstained with hematoxylin. Scale bars, 50 μm.

g Histopathology images of intestinal segments showed HSPA8-protected necroptosis-mediated SIRS shock. WT and *Rip3*$^{-/-}$ mouse intestines were subjected to H&E staining. Representative H&E-stained sections of the small intestine illustrate villus denudation and deformed mucosa.

n=3 mice for each group; scale bar, 50 μm.
