# Supplementary material for: HSPA8 acts as an amyloidase to suppress necroptosis by inhibiting and reversing functional amyloid formation
Source: Cell Res. 2023 Aug 14;33(11):851–66. doi: 10.1038/s41422-023-00859-3 (PMC10624691; doi:10.1038/s41422-023-00859-3)
Supplement: Supplementary file 5 — Supplementary information, Fig. S5 [file 41422_2023_859_MOESM5_ESM.pdf]

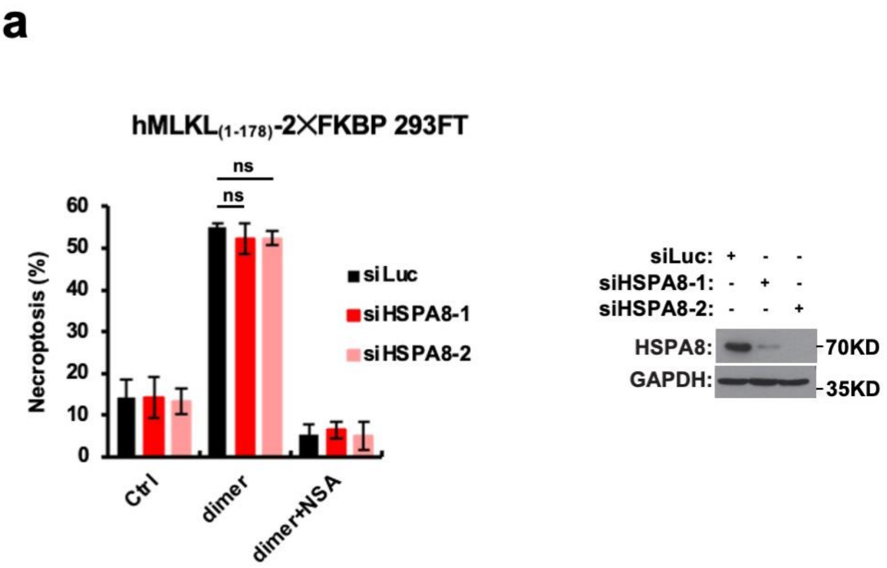

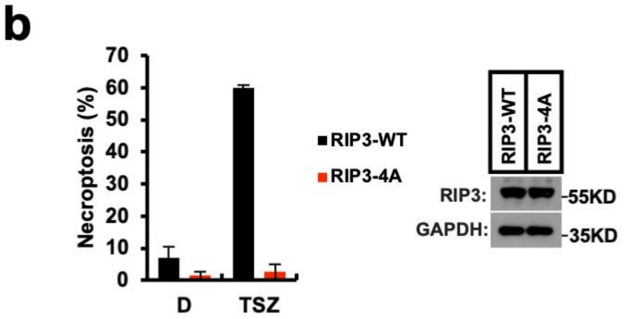

Supplementary information, Fig. S5 HSPA8 does not target MLKL.

a HSPA8 did not affect MLKL-initiated necroptosis. Thirty-six hours after transfection of siHSPA8 oligos into the hMLKL$_{1\text{-}178}$-2xFKBP-293FT cells, MLKL oligomerization was induced by adding 50 nM FKBP dimerizer (AP20187). MLKL inhibitor NSA was used to block necroptosis. The HSPA8 knockdown efficiency was tested by immunoblotting (right panel).

b Tetra-alanine substitution of the core RHIM sequence of RIP3 entirely blocked necroptosis. The Wild type and Tetra-alanine mutational RIP3 cDNAs were introduced into HeLa cells by lentivirus infection. Necroptosis was induced by treating cells with TSZ for 8 hours. Cell

viability was determined by measuring intracellular ATP levels. The data are represented as the mean ± SD of duplicate wells. The protein level of RIP3 was shown by immunoblotting analysis (right panel).

p values were determined by unpaired two-tailed Student's t-test with Welch's correction. $^{**}p < 0.01$; $^{***}p < 0.005$. *All* results are reported from one representative experiment from at least three independent repeats.
