# Supplementary material for: HSPA8 acts as an amyloidase to suppress necroptosis by inhibiting and reversing functional amyloid formation
Source: Cell Res. 2023 Aug 14;33(11):851–66. doi: 10.1038/s41422-023-00859-3 (PMC10624691; doi:10.1038/s41422-023-00859-3)
Supplement: Supplementary file 6 — Supplementary information, Fig. S6 [file 41422_2023_859_MOESM6_ESM.pdf]

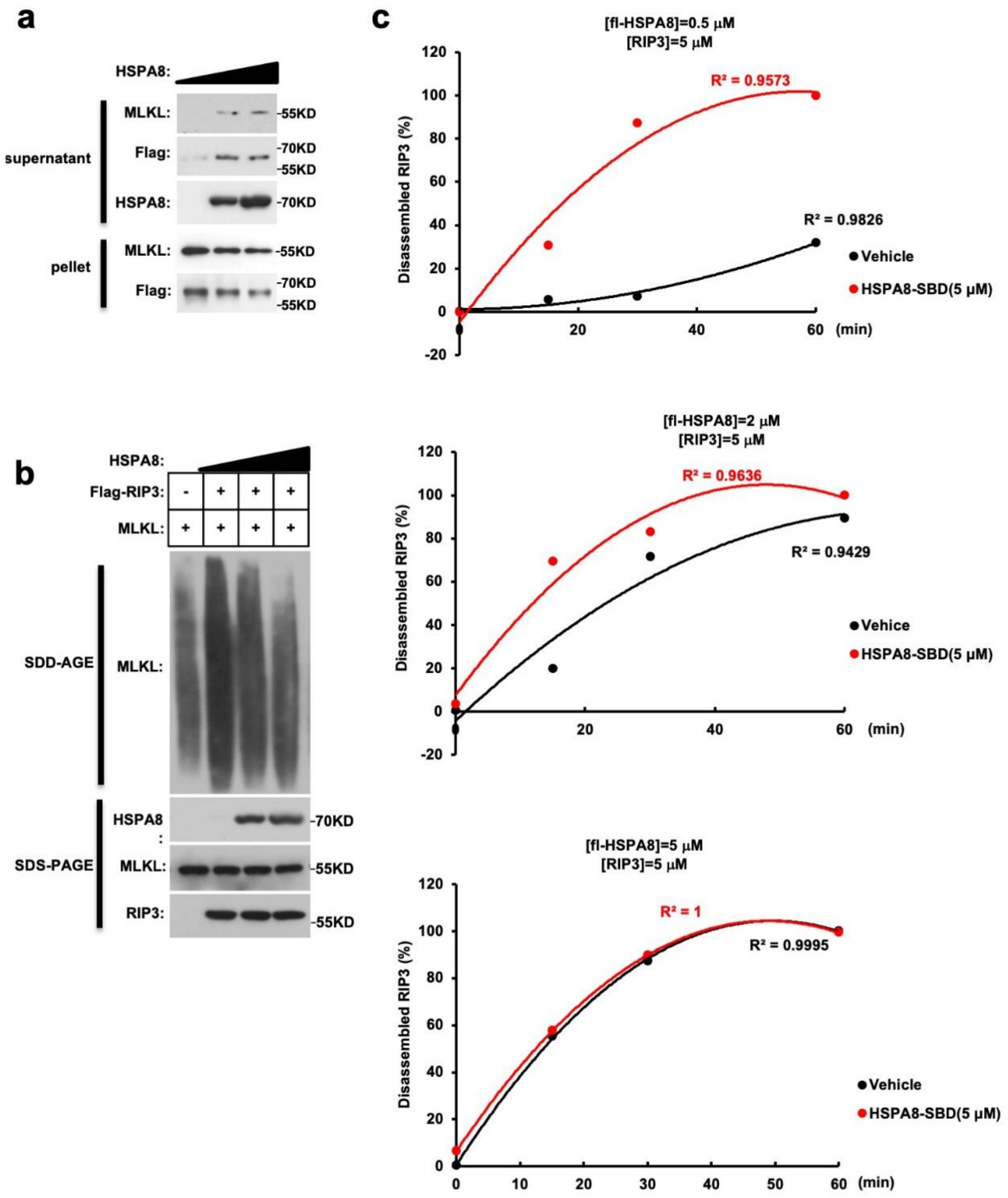

Supplementary information, Fig. S6 HSPA8 disassembles RIP3:MLKL complex (a-b), and the HSPA8 SBD domain facilitates the amyloidase activity of full-length HSPA8 by acting as a chaperone to prevent the reassembly of the disassembled RIP3 (c).

a HeLa-RIP3 cells were treated with T/S/Z for 6 hours to induce necroptosis. The whole cell lysates were subjected to immunoprecipitation using anti-Flag beads. The resulting RIP3:MLKL complexes (necrosome) were incubated with HSPA8 at 30°C for 2 hours. After centrifugation, the supernatant fraction and the pellet fraction (Flag beads) were

immunoblotted with the indicated antibodies.

b HeLa-RIP3 cells were exposed to T/S/Z for 6 hours to induce necroptosis. Immunoprecipitation of Flag-RIP3 was performed using anti-Flag beads from whole cell lysates. The immunoprecipitated complex was then incubated with HSPA8 for 30 minutes, followed by incubation with recombinant MLKL (200 nM) for 4 hours at 30°C. The oligomerized form of MLKL was subsequently detected using SDD-AGE.

c The preformed RIP3 fibrils (5 mM) were incubated with HSPA8-SBD (5 mM) and fl-HSPA8 protein (0.5 mM, 2 mM, and 5 mM, respectively) for the specified duration. Following the disassembly reaction, the soluble RIP3 and insoluble fibrils were separated through centrifugation and subjected to immunoblotting. The level of timely disassembled RIP3 in the total disassembled RIP3 was quantified using Image J software.
