# Supplementary material for: HSPA8 acts as an amyloidase to suppress necroptosis by inhibiting and reversing functional amyloid formation
Source: Cell Res. 2023 Aug 14;33(11):851–66. doi: 10.1038/s41422-023-00859-3 (PMC10624691; doi:10.1038/s41422-023-00859-3)
Supplement: Supplementary file 7 — Supplementary information, Fig. S7 [file 41422_2023_859_MOESM7_ESM.pdf]

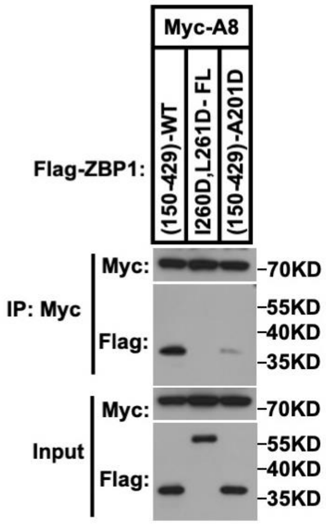

Supplementary information, Fig. S7 I260 and l261 of ZBP1 are required for HSPA8 interaction.

Mutations of the I260 or l261 of ZBP1 disrupted the interaction with HSPA8. The I260 and l261 hydrophobic residues ZBP1 were individually mutated to aspartic acid (D), and co-transfected with Myc-tagged HSPA8 into 293FT cells. The whole cell lysates were subjected to Myc-IP
