# Supplementary material for: HSPA8 acts as an amyloidase to suppress necroptosis by inhibiting and reversing functional amyloid formation
Source: Cell Res. 2023 Aug 14;33(11):851–66. doi: 10.1038/s41422-023-00859-3 (PMC10624691; doi:10.1038/s41422-023-00859-3)
Supplement: Supplementary file 8 — Supplementary information, Fig. S8 [file 41422_2023_859_MOESM8_ESM.pdf]

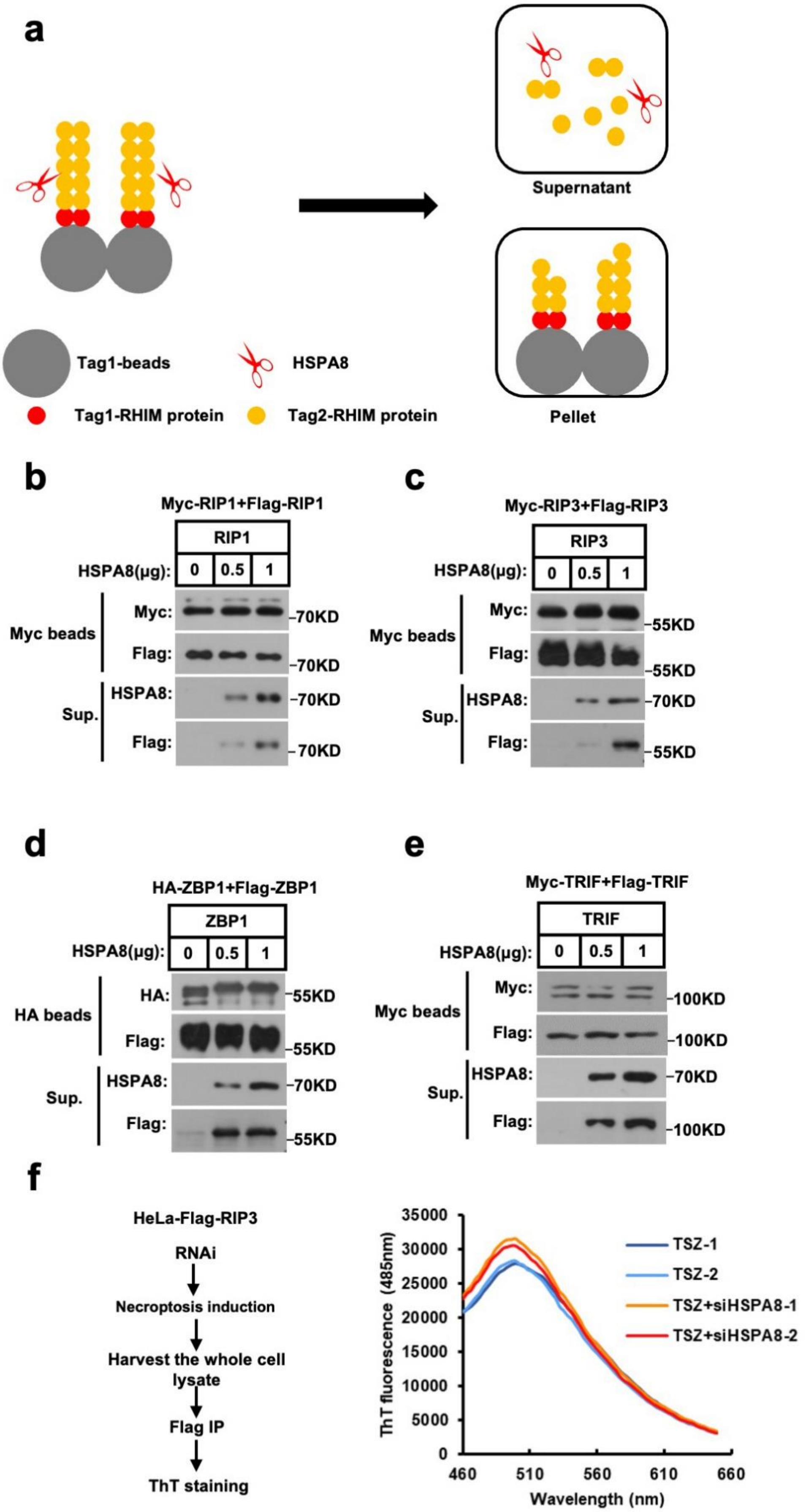
a
Supernatant
Pellet
Tag1-beads
HSPA8
Tag1-RHIM protein
Tag2-RHIM protein
b
Myc-RIP1+Flag-RIP1
RIP1
HSPA8(μg): 0 0.5 1
Myc beads
Myc: -70KD
Flag: -70KD
Sup.
HSPA8: -70KD
Flag: -70KD
c
Myc-RIP3+Flag-RIP3
RIP3
HSPA8(μg): 0 0.5 1
Myc beads
Myc: -55KD
Flag: -55KD
Sup.
HSPA8: -70KD
Flag: -55KD
d
HA-ZBP1+Flag-ZBP1
ZBP1
HSPA8(μg): 0 0.5 1
HA beads
HA: -55KD
Flag: -55KD
Sup.
HSPA8: -70KD
Flag: -55KD
e
Myc-TRIF+Flag-TRIF
TRIF
HSPA8(μg): 0 0.5 1
Myc beads
Myc: -100KD
Flag: -100KD
Sup.
HSPA8: -70KD
Flag: -100KD
f
HeLa-Flag-RIP3
RNAi
Necroptosis induction
Harvest the whole cell lysate
Flag IP
ThT staining
ThT fluorescence (485nm)
35000
30000
25000
20000
15000
10000
5000
0
460 510 560 610 660
Wavelength (nm)
TSZ-1
TSZ-2
TSZ+siHSPA8-1
TSZ+siHSPA8-2

Supplementary information, Fig. S8 HSPA8 disassembles full-length RHIM-protein oligomers.

a The Assay design of HSPA8 disassembling full-length RHIM protein aggregates as described in the **Methods**.

b, c, d, and e HSPA8 disassembled the RHIM-protein oligomers. The full-length RHIM-proteins were transfected into 293FT for 24 hours (**b** for RIP1; **c** for RIP3; **d** for ZBP1; **e** for TRIF). The harvested cells were lysed with 1% Triton-100-containing lysis buffer. The whole cell lysate was subjected to Myc-IP (or HA-IP for ZBP1 in **d)**. Then the beads were incubated with HSPA8 for 2 hours at 30 °C. The disassembled RHIM proteins were separated from the beads by centrifugation and analyzed by immunoblotting.

f ThT staining of the endogenous RIP3-containing necrosome. Left: Assay design of the purification of RIP3-containing necrosome from the necroptotic cells followed by ThT staining. HeLa-RIP3 cells were transfected with indicated siRNA oligos. Thirty-six hours later, necroptosis was induced by treating cells with T/S/Z for 10 hours. Flag-tagged RIP3 was immuno-precipitated with anti-Flag beads and eluted by Flag peptide (150μg/mL). ThT staining was detailed in the **Methods.**
